# Sulfated Aeruginosins from Lake Kinneret: *Microcystis* Bloom, Isolation, Structure Elucidation, and Biological Activity

**DOI:** 10.3390/md22090389

**Published:** 2024-08-28

**Authors:** Shira Weisthal Algor, Assaf Sukenik, Shmuel Carmeli

**Affiliations:** 1 Raymond and Beverly Sackler Faculty of Exact Sciences, School of Chemistry, Tel Aviv University, Tel Aviv 69978, Israel; shiraw1@mail.tau.ac.il; 2 The Yigal Allon Kinneret Limnological Laboratory, Israel Oceanographic & Limnological Research Institute, Migdal 49500, Israel; assaf@ocean.org.il

**Keywords:** aeruginosins, cyanobacteria, *Microcystis aeruginosa*, trypsin inhibitors

## Abstract

Aeruginosins are common metabolites of cyanobacteria. In the course of re-isolation of the known aeruginosins KT608A and KT608B for bioassay studies, we isolated three new sulfated aeruginosins, named aeruginosins KT688 (**1**), KT718 (**2**), and KT575 (**3**), from the extract of a *Microcystis* cell mass collected during the 2016 spring bloom event in Lake Kinneret, Israel. The structures of the new compounds were established on the basis of analyses of the 1D and 2D NMR, as well as HRESIMS data. Marfey’s method, coupled with HR ESI LCMS and chiral HPLC, was used to establish the absolute configuration of the amino acid and hydroxyphenyl lactic acid residues, respectively. Compounds **1**–**3** were tested for inhibition of the serine protease trypsin, and compounds **1** and **2** were found to exhibit IC_50_ values of 2.38 and 1.43 µM, respectively.

## 1. Introduction

**Figure 1:**
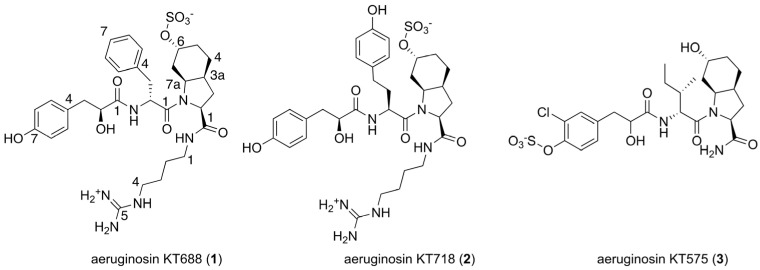
Sulfated aeruginosins isolated from *Microcystis aeruginosa* biomass collected from Lake Kinneret during the 2016 winter–spring bloom, TAU collection # IL-444.

Cyanobacteria are prolific producers of diverse groups of highly active natural products that have been summarized over the years in many reviews [1,2,3,4]. Cyanobacteria in marine and freshwater environments produce, in many cases, the same families of compounds, i.e., linear peptolides such as the symplostatins [5] and mirabimides [6]; cyclohexamides such as the venturamides [7] and the microcyclamides [8]; cyclic peptides such as the pahayoklides [9] and the schizotrins [10]; and cyclic depsipeptolides such as the lyngbyastatins [11] and the micropeptins [12]. Moreover, in some cases, similar compounds were isolated from marine sponges and freshwater cyanobacteria, i.e., the mozamides, which were isolated from a Theonellid sponge [13], resemble the anabaenopeptins that were isolated from freshwater *Microcystis*, *Anabaena,* and *Nostoc* spp. [14], and the dysinosins that were isolated from an Australian sponge of the family Dysideidae [15] are similar to the aeruginosins, which are frequently isolated from freshwater cyanobacteria [16]. They are a group of linearly modified peptides typically composed of four building blocks, where the *N*-terminus is an α-hydroxy acid unit, followed by an aromatic or aliphatic amino acid residue, which is connected to the key modified amino acid motif, 2-carboxy-6-hydroxy-octahydroindole (Choi), 5-oxy-Choi, or proline derivative, and terminated with an arginine derivative [17]. The aeruginosins were isolated from water–bloom-forming cyanobacteria of the genus *Microcystis*, *Planktothrix*, *Nodularia,* and *Nostoc* and symbiotic cyanobacteria in sponges [18]. To date, 86 metabolites related to this family of compounds have been fully characterized, and most of them inhibit serine proteases of the trypsin clade, while an additional 46 have been characterized by LC–MS/MS techniques (see Table S1 in Supplementary Materials) [17,18]. Despite the relatively small size of these modified peptides, the structure elucidation of the aeruginosins is not straightforward. They usually appear as a mixture of *cis* and *trans* rotamers around the bond contacting the second and third sub-units, of varying percentages governed by the structure of the subunits, solvent, and temperature [19]. In the process of isolating the known aeruginosins KT608A and KT608B [19,20] for bioassay studies from the 2016 spring *Microcystis* bloom material (dominant by the *Microcystis aeruginosa* strain marked Mic B due to its dominant pigmentation, brown color) [21,22], we isolated, among other groups of compounds [23], three new sulfated aeruginosins named aeruginosins KT688 (**1**), KT718 (**2**), and KT575 (**3**) (Figure 1). Herein, we describe the structure elucidation and biological activity of the new compounds **1**–**3**.

## 2. Results

The dried aqueous methanolic extract of the *Microcystis* bloom material was separated by flash chromatography on a reversed-phase C_18_ MPLC column, followed by gel filtration on a Sephadex LH-20 column. The sulfated aeruginosin-containing fraction (determined by the characteristic ^1^H NMR spectrum and LCMS) was repeatedly separated on a reversed-phase HPLC column to afford the three new aeruginosins KT688 (**1**), KT718 (**2**), and KT575 (**3**).

**Figure 2:**
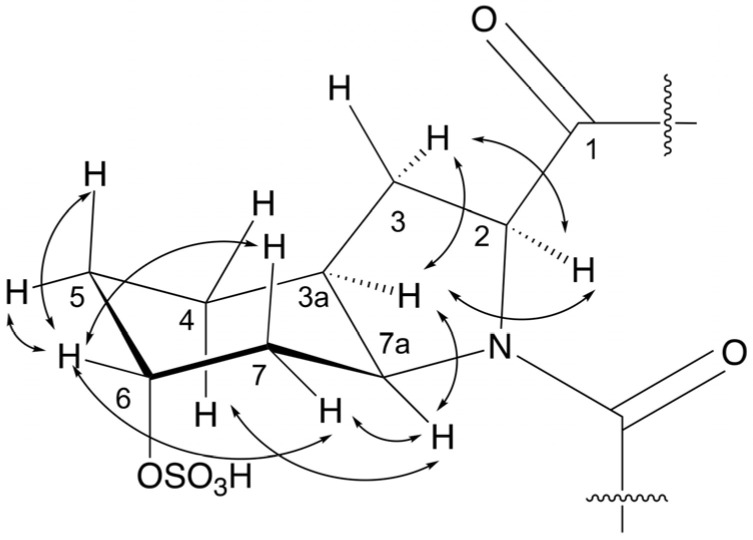
Key NOE correlations of the *trans* rotamer of the Choi-sulfate moiety of **1**.

Aeruginosin KT688 (**1**) is a white solid that exhibited a positive HRESIMS molecular cluster ion [M+Na]^+^ at *m*/*z* 711.2802, corresponding to the molecular formula of C_32_H_44_N_6_NaO_9_S^+^ and 14 degrees of unsaturation. When **1** was dissolved in DMSO-*d*_6_, it appeared in the ^1^H and ^13^C NMR spectra as a 4:1 mixture of two rotamers. Both were fully characterized (Table 1 and Table S2), but for the simplicity of the discussion, only the major one is described below. The ^1^H NMR spectrum of the major species of **1** in DMSO-*d*_6_ (Table 1) presented signals of six exchangeable protons: two protons of secondary amide (δ_H_ 7.62 d and 7.68 t); a hydroxy proton (δ_H_ 5.75 brm); and NH protons of a guanidinium motif (δ_H_ 8.58 and 8.69 brs, 3H). The aromatic region of the ^1^H spectrum (Table 1) presented overlapping signals indicating the presence of a phenyl moiety (δ_H_ 7.10 d; 7.14 t; 7.23 t, integrated to 2H, 1H, and 2H, respectively), as well as doublet signals of a pair of signals of a *para*-substituted phenol (δ_H_ 6.83 d; 6.56 d, 2Hs each). Five protons next to a heteroatom (δ_H_ 3.99 dd; 4.64 q; 4.05 t; 4.29 brs; 3.66 dt; and 3.02 m) and several aliphatic protons in the 1.3−1.6 ppm region. The ^13^C NMR spectrum of **1** in DMSO-*d*_6_ (Table 1) revealed three carboxy carbons (δ_C_ 173.2, C 169.0 C and 171.5, C), a guanidinium carbon (δ_C_ 157.5), 12 signals of aromatic sp^2^ carbons consistent with a para-substituted phenol (δ_C_ 128.4, C, 130.3, 2 × CH, 114.8, 2 × CH, and 155.8, C) and a phenyl residue (δ_C_ 138.3, C 129.3, 2 × CH, 128.1, 2 × CH, and 126.3, CH), two sp^3^ carbons next to oxygen (δ_C_ 72.3, CH and 70.9, CH), five sp^3^ carbons adjacent to nitrogen (δ_C_ 59.9, CH; 54.3, CH; 51.5, CH; 40.4, CH_2_; 38.1, CH_2_), and nine aliphatic carbons (single methine and eight methylenes) between 40 and 19 ppm. Interpretation of the 1D and 2D NMR data (^1^H, ^13^C, COSY, TOCSY, ROESY, HMQC, and HMBC) enabled the assignment of two sets of four residues: *p*-hydroxyphenyl lactic acid (Hpla), Phe, Choi-6-sulfate, and agmatine (Agm) (Table 1 and Table S2 in Supplementary Materials). The assignment of the sulfate to Choi-6-position was supported by a good matching of its ^1^H and ^13^C NMR chemical shifts (δ_H_ 4.35, brs and δ_C_ 70.9 in **1**; δ_H_ 4.29, brs and δ_C_ 70.8 in aeruginosin GE766 versus δ_H_ 3.90, brs and δ_C_ 64.1, in aeruginosin GE686, which harbor an L-Choi-6-OH residue) and with the ^13^C NMR chemical shifts of the whole residue of aeruginosin GE766 (see the comparison in Table S3) [24]. HMBC and ROESY correlations allowed the assembly of the four residues into the linear structure Hpla-Phe-Choi-6-sulfate-agmatine. The NOEs of the major isomer, Phe-H-2 with Choi-H-7eq and Choi-H-7a, assigned it as the *trans* rotamer, while those of Phe-H-2 with Choi-H-2 of the minor isomer assigned it as the *cis* rotamer. The ^1^H and ^13^C NMR chemical shifts of the Choi moiety of the **1** minor *cis* rotamer differed from those in the aeruginosins KT608A and KT608B [19] major *cis* rotamers (see Table S4). The substantial chemical shift differences in the cyclohexane ring of the Choi moiety (see Table S4) suggest that either the substitution of Choi-6-OH with a sulfate moiety and/or different configurations of the Choi-chiral centers triggered the shifting of the equilibrium between the two rotamers toward the *trans* rotamer in **1**. The relative configuration of the chiral centers of the Choi-sulfate moiety was established based on the NOE configurations within the Choi moiety (Figure 2), determining that H-2, H-3a, and H-7a are on the same plan of the five-membered ring, H-4a, H-7pe, and H-7a are on the same plan of the cyclohexane ring, and H-6 is equatorial, suggesting that the Choi-sulfate moiety is either L or D [24]. The absolute configurations of the two amino acid residues of **1** was determined by applying Marfey’s method [25] (L-FDAA) in conjugation with HR ESI LCMS, which revealed the presence of L-Choi (by comparison with the derivative obtained from the hydrolysis of aeruginosin GE686 [24]) and D-Phe moieties. Chiral-phase HPLC chromatography of the hydrolysate-ether extract of **1** with D/L-Hpla established it as L-Hpla residue in 1. Thus, the structure of aeruginosin KT688 (**1**) was established as L-Hpla-D-Phe-L-Choi-6-sulfate-agmatine.

Aeruginosin KT718 (**2**) was isolated as a white solid that exhibited a positive HRESIMS molecular cluster ion at *m*/*z* 741.2906 [M+Na]^+^ corresponding to the molecular formula C_33_H_46_N_6_NaO_10_S^+^ and 14 degrees of unsaturation. Both its ^1^H and ^13^C NMR spectra in DMSO-*d*_6_ presented two sets of peaks at a ratio of 2:1, suggesting its existence as a mixture of rotamers. The ^1^H NMR and ^13^C NMR spectra of **2** in DMSO-*d*_6_ (Table 2 and Table S5) show similar signals as compound **1,** except for chemical shifts and changes in the signals of the aromatic amino acid. Analysis of the major rotamers by 2D NMR (HSQC, HMBC, COSY, TOCSY, and ROESY) (Table 2) established that compound **2** comprised four units, three of which were the same as those of compound **1**: Hpla, Choi-6-sulfate, and agmatine. The fourth, an aromatic residue, was constructed based on COSY correlations of an amide NH (δ_H_ 7.71, d) with an α-methine (δ_H_ 4.49, dt, H-2), which in turn correlated to an aliphatic methylene (δ_H_ 1.77 and 1.69, m, H_2_-3) and further to a benzylic methylene (δ_H_ 2.38, m, H_2_-4), and of the aromatic methine protons (δ_H_ 6.94, d, 2H, H-6,6′), which presented a correlation with another aromatic methine protons (δ_H_ 6.65, d, 2H, H-7,7′). HSQC correlations allowed the assignment of carbons to the latter protons (Table 2). HMBC correlations of H-2 with an amide carbonyl carbon (δ_C_ 169.7, C-1) assigned it as the carbonyl of this amino acid residue. HMBC correlations of H_2_-4 with a quaternary aromatic carbon (δ_C_ 131.6, C-5) and C-6,6′ (δ_C_ 129.1) assigned the first as the carbon linking the aromatic ring to the aliphatic portion of this residue. HMBC correlations of H-6,6′,7,7′ with a phenolic carbon (δ_C_ 155.6, C-8) culminated in the assignment of the signals of this amino acid unit and identified it as homotyrosine (Hty). HMBC correlations of the Hpla carboxyl (δ_C_ 173.4) with Hty-2-NH, together with NOE correlations of Hty-H-2 with Choi-H-7ax and H-7a (which also assigned the major rotamer as the *trans* rotamer) and NOE correlation of Choi-2-H with Agm-1-NH, established the planar structure of the sequence Hpla-Hty-Choi-6-sulfate-agmatine. Finally, derivatization of the peptide hydrolysate with L-FDAA reagent [21] revealed the presence of L-Choi and L-Hty, and the analysis on the Chiral-phase HPLC column of the hydroxy acid residue established the absolute configuration of Hpla as L, therefore establishing the structure of aeruginosin KT718 (**2**) as L-Hpla-L-Hty-L-Choi-6-sulfate-Agm.

Aeruginosin KT575 (**3**) was isolated as a yellowish powder exhibiting a negative HRESIMS molecular cluster ion at *m*/*z* 574.1647/576.1615 (3:1) [M–H]^−^, characteristic of a chlorine-containing molecule, and corresponding to a molecular formula of C_24_H_33_N_3_ClO_9_S^−^ and nine degrees of unsaturation. The positive HRESIMS gave a desulfated cluster ion at *m*/*z* 496.2213/498.2208 [M-SO_3_+H]^+^ characteristic of sulfate ester [26]. The ^1^H NMR spectrum of **3** in DMSO-*d*_6_ (Table 3) presented signals indicative of two rotamers in a ratio of 10:1 (only the major was fully analyzed), with five exchangeable protons (a secondary amide, NH 7.37 d; two hydroxyl protons, δ_H_ 4.53 d, and 5.98 d; and two primary amide protons, δ_H_ 7.29 d, and 6.83 d), three aromatic protons (δ_H_ 7.22 d, 7.07 d and 7.45 dd), five protons next to heteroatom (δ_H_ 4.09 m; 4.55 dd; 4.14 dd; 3.90 brs; and 4.05 brt), several aliphatic protons, and two methyl groups (δ_H_ 0.88 t and 0.65 d). The ^13^C NMR spectrum of **3** in DMSO-*d*_6_ (Table 3) revealed three carboxyl carbons (δ_C_ 173.6, 168.7 C and 172.3, C), six signals of aromatic sp^2^ carbons (δ_C_ 121.3, 128.8, 123.8, 148.0, 130.5 and 134.0), two sp^3^ carbons next to oxygen (δ_C_ 71.9, CH and 64.1, CH), three sp^3^ carbons adjacent to nitrogen (δ_C_ 59.7, CH; 54.0, CH and 51.8, CH), ten aliphatic sp^3^ carbons (four methines and six methylenes) between 40 and 19 ppm, and two methyl groups (δ_C_ 14.1 and 12.0). Interpretation of the 1D and 2D NMR data (^1^H, ^13^C, COSY, TOCSY, ROESY, HMQC, and HMBC) enabled the assignment of the NMR signals of **3** (Table 3). The three aromatic protons [δ_H_ 7.45, d (8.9); 7.22, d (2.1); 7.07, dd (8.9, 2.1)] presented coupling constants and COSY correlations indicative of a 1,2,4-trisubstituted phenyl moiety. HMBC correlations (Table 3) of the latter protons and the aliphatic protons (δ_H_ 2.93 and 2.70) with the aromatic carbons indicated that the carbon at δ_C_ 134.0 bridges the aromatic moiety and the aliphatic substituent and that the aromatic methine carbons at δ_C_ 130.5 and 128.8 are directly connected to it. The correlations of the aromatic protons with the quaternary carbon at δ_C_ 148.0 proved its location *para* to the aliphatic substitution and that resonating at δ_C_ 123.8 is situated at the *meta* position. The chemical shift of the oxygenated quaternary carbon at δ_C_ 148.0 fitted that of sulfated phenol ester and that of quaternary carbon at δ_C_ 123.8, a chlorine-bearing aromatic carbon as in aeruginosin 89-B (Table S6) [27]. COSY correlations of the protons of the benzylic methylene (δ_H_ 2.92, dd and 2.70, dd ppm) with the oxymethine proton (δ_H_ 4.09) and of the latter with the hydroxyl proton (δ_H_ 5.98, d) identify the moiety as *m*-chloro-*p*-Hpla-sulfate. Finally, HMBC correlations of the protons at δ_H_ 5.98, 4.09, 2.92, and 2.70 with the carboxyl carbon at δ_C_ 172.3 ended the assignment of the substituted-Hpla moiety. The second subunit was identified as an isoleucyl moiety. The sequence of COSY correlations from Ile-H-2 (δ_H_ 4.55) through H_3_-5 and H_3_-6 (Table 3) assigned an isoleucine proton spin system. The HMBC correlation of Ile-H-2 with a carboxyl carbon at δ_C_ 168.7 assigned it to the Ile moiety. COSY correlations (Table 3) established the connectivity of Choi-H-2–H_2_-3–H-3a–H-7a–H_2_-7–H-6–H_2_-5, the connectivity of H-3a–H_2_-4, and the connectivity of H-6–6-OH. The connectivity between H_2_-5 and H_2_-4 was proved by the TOCSY correlation of H-6 and H-4 at δ_H_ 2.02. HMBC correlations of H-2 and the primary amide protons (δ_H_ 7.29 and 6.83) with the carboxy carbon at δ_C_ 173.6 assigned the latter as C-1 of the amino acid moiety. The HMBC correlation of H-7a with the amino-methine at δ_C_ 59.7 (C-2) suggested a nitrogen bridge between C-2 and C-7a, assigning the moiety as Choi. The assigned protons and carbons of the Choi residue presented an excellent match to the chemical shifts of the same unit in aeruginosin DA495B (Table S7) [28]. HMBC correlations of Hpla-C-1 with Ile-2-NH, NOE correlations of Ile-2-NH with Hpla-2-OH, and Ile-2-H with Choi-H-7a assigned the consecutive sequence structure of 6-Cl-Hpla-7-sulfate-2-Ile-Choi-amide. The NOE between the protons Choi-7a and Ile-2 assigned the major rotamer as *trans.* Applying Marfey’s method (L-FDAA) [25] revealed the presence of L-Choi and D-Ile moieties in **3**. However, we could not distinguish D-Ile from D-*allo*Ile due to their similar retention times under the HPLC conditions we applied. However, this could be circumvented based on the characteristic carbon chemical shifts of C-5 and C-6 of the Ile and *allo*Ile. The chemical shifts of Ile-C-5 and C-6 in **3** (δ_C_ 12.0 and 14.1), respectively, were similar to those of D-*allo*Ile in aeruginosins 98-C (δ_C_ 11.6 and 13.8) and 101 (δ_C_ 11.8 and 13.6) [27] and different from those of L-Ile in aeruginosin BH604 (δ_C_ 10.9 and 15.6) [29]. The absolute configuration of the 3-Cl-Hpla was not determined. Based on these arguments, the structure of aeruginosin KT575 was established as **3**.

The biological activity of **1–3** was examined against the serine protease trypsin. Aeruginosin KT688 (**1**) was found to inhibit trypsin with an IC_50_ values of 2.38 +/− 0.59 µM, while aeruginosin KT718 (**2**) had an IC_50_ valu of 1.43 +/− 0.25 µM. Aeruginosin KT575 (**3**) was found to be inactive at a concentration of 1.7 mM, as expected, due to the absence of a C-terminal agmatine derivative.^2^ Moreover, the latter results reinforce the earlier findings that aeruginosins that contain the Choi-6-sulfate moiety inhibit trypsin-type proteases similar to those containing the Choi moiety [24,27].

## 3. Materials and Methods

### 3.1. General Experimental Procedure

Optical rotation values were obtained on a Jasco P-1010 polarimeter (JASCO Corporation, 2967-5, Ishikawa-machi, Hachioji-shi, Tokyo, Japan) at the sodium D line (589 nm). UV spectra were recorded on an Agilent 8453 spectrophotometer (Agilent, Santa Clara, CA, USA). IR spectra were recorded on a Bruker Tensor 27 FT-IR instrument (Bruker, Billerica, MA, USA). NMR spectra were recorded on Bruker Avance III spectrometers (Bruker, Karlsruhe, Germany) at 500.13 or 400.17 MHz for ^1^H and 125.76 or 100.63 MHz for ^13^C; chemical shifts were referenced to TMS δ_H_ and δ_C_ = 0 ppm. COSY-45, gTOCSY, gROESY, gHSQC, and gHMBC spectra were recorded using standard Bruker pulse sequences. ESI low- and high-resolution mass spectra and MS/MS spectra were recorded on a Waters (Waters, Milford, MA, USA) Xevo G2-XS QTOP instrument equipped with Acquity Hi Class UPLC (binary solvent manager) with an FTN sample manager, column manager, and PDA detector, using a 2.1 × 50 mm BEH C18 (1.7 mm) column and a flow rate of 0.1–0.3 mL/min. HPLC separations were performed on an Agilent 1100 Series HPLC system (Agilent, Santa Clara, CA, USA). The kinetic measurement of the absorbance intensity was measured on a TECAN Infinite 200 Pro multiplate reader (Tecan, Grodig, Austria).

### 3.2. Biological Material

*Microcystis* biomass, TAU collection number IL-444, was collected in February 2016 from Lake Kinneret, Israel. The cell mass was frozen and lyophilized. A sample of the cyanobacteria is deposited at the culture collection of Tel Aviv University. The *Microcystis* bloom was dominant in *Microcystis aeruginosa* strain B due to its dominant pigmentation and brown color [21,22].

### 3.3. Isolation Procedure

The freeze-dried cell mass (515 g) was extracted with 7:3 MeOH:H_2_O (3 × 4 L). The solvent was evaporated to dryness (45 g). Aliquots of the extract were fractionated (10 g in each separation) on an octadecyl-silica flash column (YMC-GEL, ODS, 120 Å, 4.4 × 6.4 cm), with an increasing concentration of MeOH in H_2_O. Fraction A9 (1 g), eluted from the flash column with 8:2 MeOH:H_2_O, was separated on a CombiFlash EZPrep C-18 column (Teledyne ISCO, 15.5 gr HP C18, 250 mg loaded in each separation) using linear gradient conditions from 5% to 100% MeCN (at an increment rate of 1% MeCN/min and a flow rate of 7 mL/min), resulting in 15 fractions. The fractions were analyzed by NMR and MS and merged into the final 10 fractions (B1–B10). Selected fractions from the above were dissolved (20:80 MeCN:H_2_O) and separated on a semipreparative HPLC RP-18 column (YMC Pack ODS-AQ, 5 µm, 250 × 10 mm). Fraction B1 (24 mg) was separated under gradient conditions, from 8:1 to 2:1, 0.05% aqueous formic acid/MeCN, at an increment rate of 0.3% MeCN/min and a flow rate of 2.5 mL/min, to yield aeruginosin KT575 (**1**, 1.8 mg, R_t_ 45.2 min, 3.5 × 10^−4^% yield). Fraction B3 (41 mg) was separated under isocratic conditions 7:1, 0.05% aqueous formic acid/MeCN, 2 mL/min (YMC Pack ODS-AQ, 5 µm, 250 × 10 mm) to yield aeruginosin KT718 (**2**, 1.5 mg, R_t_ 29.7 min, 2.9 × 10^−4^% yield from dry cell weight) and aeruginosin KT688 (**3**, 2.2 mg, R_t_ 52.4 min, 4.3 × 10^−4^% yield).

### 3.4. Physical Data of the Compounds

*Aeruginosin KT688* (**1**): [α]_D_^22^ = −8.8 (c 0.0011, MeOH); UV (MeOH) λ_max_ (log ε) 202 (4.15), 222 (3.91), 277 (3.03) nm; IR (ATR, Diamond) v_max_ 3357, 2943, 2833, 1595, 1450, 1412, 1364, 1259, 1112, 1021 cm^−1^; For ^1^H and ^13^C NMR data, see Table 1 and Table S2; HRESIMS [M+Na]^+^, *m*/*z* 711.2802 (calc. for C_32_H_44_N_6_NaO_9_S^+^, *m*/*z* 711.2788).

*Aeruginosin KT718* (**2**): [α]_D_^22^ −14.0 (c 0.0019, MeOH); UV (MeOH) λ_max_ (log ε) 202 (4.15), 222 (3.79), 277 (2.93) nm; IR (ATR, Diamond) v_max_ 3345, 2946, 2833, 1594, 1516, 1450, 1353, 1239, 1111, 1021 cm^−1^; For ^1^H and ^13^C NMR data, see Table 2 and Table S5; HRESIMS [M+Na]^+^, *m*/*z* 741.2906 (calc. for C_33_H_46_N_6_O_10_NaS^+^, *m*/*z* 741.2894).

*Aeruginosin KT575* (**3**): [α]_D_^22^ = −15.3 (c 0.0016, MeOH); UV (MeOH) λ_max_ (log ε) 202 (4.15), 223 (4.03), 276 (3.22) nm; IR (ATR, Diamond) v_max_ 3345, 2944, 2833, 1633, 1529, 1492, 1413, 1238, 1111, 1022 cm^−1^; For ^1^H and ^13^C NMR data, see Table 3; HRESIMS [M–H]^−^, *m*/*z* 574.1647 (calc. for C_24_H_33_^35^ClN_3_O_9_S^−^, *m*/*z* 574.1626).

### 3.5. Determination of the Absolute Configuration of the Amino Acids by Marfey’s Method [30]

Compounds **1**–**3** and an authentic sample of aeruginosin GE686 [24] were hydrolyzed in 6 N HCl (1 mL). The reaction mixture was maintained in a sealed glass bomb at 110 °C for 20 h. The acid was removed in vacuo, and the residue was suspended in 250 µL of H_2_O. A solution of 1-fluoro-2,4-dinitrophenyl-5-L-alanine amide (FDAA) in acetone (0.03 M, 1:1.1 mole-equivalent per each amino acid in the peptide) and NaHCO_3_ (1 M, 20 µL per each amino acid) was added to the reaction vessel. The reaction mixture was stirred at 70 °C for 3 h in the dark. HCl (2 M, 10 µL per amino acid) was added to the reaction vessel, and the solution was evaporated in vacuo. The samples of L-FDAA derivatives were analyzed by ESI LC–MS. The analysis was performed on a Waters (USA) Xevo G2-XS QTOP instrument equipped with Acquity Hi Class UPLC (binary solvent manager) with an FTN sample manager, column manager, and PDA detector, using a 2.1 × 50 mm BEH C18 (1.7 mm) column and a flow of 0.1 or 0.2 mL/min. The mobile phase compositions were (A) water containing 0.5% acetic acid and (B) MeCN containing 10% MeOH. The elution gradient was as follows: 1 min of 100% A, a linear gradient to 60% B over 30 min, and then recycled by a linear gradient to 5% B over 10 min and to 100% A over an additional 5 min. Samples of 10 µL were injected, and the flow rate was 0.1 or 0.2 mL/min. The UV detector was set to 340 nm, and the mass spectrometer was operated in both negative and positive ion modes, scanning between 100 and 1000 mass units. The interpretation of the data was conducted after the run on both positive and negative ion modes using Waters MassLynx software (v.4.1).

### 3.6. Determination of Absolute Configuration of the Hydroxy Acids

A total of 0.25 mg portions of compounds **1**–**2** were dissolved in 6 N HCl (1 mL), and the reaction mixture was then placed in a sealed glass bomb at 110 °C for 20 h. The ethereal extract of the acid hydrolysate of **1**–**3** was removed in vacuo, and the residue was dissolved in MeOH (1 mL). The MeOH solution was analyzed on a Chiral Technologies Inc. (West Chester, PA, USA) CHIRALPAK™ AD-H, LC Stationary Phases, 250 × 4.6 mm flow rate, 0.1 mL/min, UV detection at 230 nm, isocratic elution, 18% isopropyl alcohol in hexane. The Hpla residues from the aeruginosins were compared with the L,D-Hpla standard.

### 3.7. Protease Inhibition Assays

The samples for biological assays were dissolved in ethanol at a concentration of 1 mg/mL and further diluted with Tris buffer to the desired concentrations. The samples were tested for inhibition of trypsin (Cat# T1426, Sigma-Aldrich, St. Louis, MO, USA). The assay was performed in a Tris buffer (50 mM Tris-HCl, 100 mM NaCl, and 1 mM CaCl_2_ at pH 7.5). Benzoyl-L-arginine-*p*-nitroanilide hydrochloride (BAPNA Cat# 03,310 Chem-Impex Int’l Inc., Wood Dale, IL, USA), the trypsin substrate was dissolved at 1 mg/mL in 1:9 EtOH/buffer. The enzyme was dissolved in a buffer at 1 mg/mL. To each well were added 100 µL of buffer, 10 µL of enzyme, and 10 µL of sample. The plate was placed in the spectrophotometer at 37 °C. After 5 min, 100 µL of substrate solution was added to each well, and the plate was placed in the spectrophotometer for the kinetic measurement of the absorbance intensity over 10 min at a wavelength of 405 nm.

## 4. Conclusions

We have been monitoring the seasonal blooms of cyanobacteria in Lake Kinneret for almost three decades [19,31,32]. Over the years, the spring *Microcystis* bloom was shifted from green to brown in color (dominant by the *Microcystis aeruginosa* strain marked Mic B due to its dominant pigmentation, brown color) [21,22]. The brown bloom material contains a large amount of the aeruginosins KT608A and KT608B, which we use for an ongoing study on their ecological role. While isolating the aeruginosins for our study, we noticed several unknown higher masses that easily lost 80 mass units in the LC–MS/MS of a fraction that eluted earlier of the aeruginosins KT608A and KT608B. The IC_50_ values of aeruginosin KT688 (**1**) and aeruginosin KT718 (**2**) for trypsin are comparable with those of aeruginosins KT608A and KT608B [19]. In many cases (Table S1), *Microcystis* blooms produce an array of aeruginosins and other modified small peptides rather than a single active compound, leaving us with an open question of why the cyanobacterium invests resources in producing an array of similar compounds with similar activities.

## Figures and Tables

**Table 1:** NMR Data of the Major *trans* Rotamer of Aeruginosin KT688 (**1**) in DMSO-*d*_6_^a^.

Position	δ_C_ mult.	δ_H_ mult. (*J* in Hz)	HMBC Correlation	COSY Correlation	TOCSY Correlation	ROESY Correlation
Hpla 1	173.2, C		Phe-2,2-NH, Hpla-2,3a,3b			
2	72.3, CH	3.99, dd (7.9, 3.2)	Hpla-3	Hpla-3a,3b	Hpla-3a,3b	Phe-2-NH, Hpla-3a,3b,5,5′
2-OH		5.75, brm				
3a 3b	39.6, CH_2_	2.81, dd (14.1, 3.5) 2.55, dd (14.1, 8.0)	Hpla-5,5′	Hpla-2,3b Hpla-2,3a	Hpla-2,3b Hpla-2,3a	ChoiSul-2, Hpla-2,3b, 5,5′ Hpla-2,3a, 5,5′
4	128.5, C		Hpla-2,3a, 3b,6,6′,			
5,5′	130.6, CH × 2	6.98, d (8.4)	Hpla-3a, 3b,5′,5	Hpla-6,6′	Hpla-6,6′	Hpla-2, 2.81, 2.55
6,6′	114.9, CH × 2	6.64, d (8.4)	Hpla-6′,6	Hpla-5,5′	Hpla-5,5′	Phe-5,5′
7	155.9, C		Hpla-5,5′, 6,6′			
Phe 1	169.0, C		Phe-2,2-NH,3a,3b, ChoiSul-7a			
2	51.5, CH	4.64, q (7.1)	Phe-2-NH, 3a,3b	Phe-2-NH, 3a,3b	Phe-2-NH, 3a,3b	Phe-2-NH, 3a,3b,5,5′, ChoiSul-7eq,7a
2-NH		7.62, d (7.1)		Phe-2	Phe-2,3a, 3b	Phe-2,3a, 3b, Hpla-2
3a 3b	38.3, CH_2_	2.85, dd (13.2, 6.4) 2.76, dd (13.2, 8.1)	Phe-2-NH, 5,5′	Phe-2,3b Phe-2,3a	Phe-2, 2-NH,3b Phe-2, 2-NH,3b	Phe-2, 2-NH,3b,5,5′, ChoiSul-7a Phe-2, 2-NH,3b,5,5′, ChoiSul-7a
4	136.4, C		Phe-2,3a, 3b,6,6′			
5,5′	129.7, CH × 2	7.10, d (7.3)	Phe-3a,3b 5′,5	Phe-6,6′,	Phe-6,6′,	Phe-2,3a, 3b, ChoiSul-2,7a
6,6′	128.4, CH × 2	7.26, t (7.3)	Phe-6′,6	Phe-5,5′,7	Phe-5,5′,7	ChoiSul-2
7	126.7, CH	7.20, t (7.3)	Phe-5,5′	Phe-6,6′	Phe-6,6′	
ChoiSul 1	171.5, C		Agm-1-NH,1, ChoiSul-2,3pa			
2	59.9, CH	4.05, t (9.1)	ChoiSul-7a	ChoiSul-3pa, 3pe	ChoiSul-3pa, 3pe,3a,7pe, 7a	Agm-1-NH, Phe-5,5′,6,6′, ChoiSul-3pe, 3a
3pe ^b^ 3pa ^b^	30.6, CH_2_	1.89, m 1.66, m	ChoiSul-2,7a	ChoiSul-3pa, 3a ChoiSul-3pe, 3a	ChoiSul-3pa, 3a,5a, ChoiSul-3pe, 3a	ChoiSul-2,3pa,3a ChoiSul-3pe
3a	35.8, CH	1.58, m	ChoiSul-7a	ChoiSul-3pe, 3pa,4a,7a,		ChoiSul-3pe,7a
4a 4b	19.4, CH_2_	1.71, m 1.29, m		ChoiSul-3a,4b ChoiSul-3a,4a		ChoiSul-7a
5a 5b	23.7, CH_2_	1.70, m 1.28, m		ChoiSul-5b,7pe ChoiSul-5a	ChoiSul-5b ChoiSul-5a	
6	70.9, CH	4.29, brs	ChoiSul-5b	ChoiSul-5a,5b, 7pe,7pa	ChoiSul-5pe, 5b,7pe,7pa, 7a	Phe-3a, ChoiSul-5a,5b,7pe,7pa
7pe ^b^ 7pa ^b^	31.3, CH_2_	2.27, m 1.49, m		ChoiSul-5pe, 7pa ChoiSul-7pe	ChoiSul-3pe, 7pa ChoiSul-3pe, 7pe	Phe-2, ChoiSul-6,7pa,7a, ChoiSul-6,7pe
7a	54.3, CH	3.66, dt (11.1, 5.8)	ChoiSul-3pe,3a	ChoiSul-3a, 7pe,7pa	ChoiSul-2,3a, 3pa,6,7pe, 7pa	Phe-2,5,5′, ChoiSul-3a,4a,7pe
Agm-1-NH		7.68, t (5.0)		Agm-1	Agm-1, 1.41	ChoiSul-2, Agm-1, 2
1	38.1, CH_2_	3.02, m	Agm-1-NH		Agm-1-NH,2,4-NH	Agm-1-NH,2,4-NH
2	25.9, CH_2_	1.41, m	Agm-3,4		Agm-1-NH	
3	26.3, CH_2_	1.41. m	Agm-2,4		Agm-4-NH	Agm-1-NH,4-NH
4	40.4, CH_2_	3.02, m		Agm-1-NH, 12	Agm-1-NH, 2	Agm-2,3,4-NH,
4-NH		8.58, brs		Agm-4	Agm-3,4	Agm-3,4
5	157.5, C		Agm-4			
5-NH, NH_2_		8.58, brs 8.69, brs		Agm-4	Agm-3,4	Agm-3,4

^a^ 500 MHz for ^1^H, 125 MHz for ^13^C. ^b^ pe: pseudo-equatorial, pa: pseudo-axial.

**Table 2:** NMR Data of the Major *trans* Rotamer of Aeruginosin KT718 (**2**) in DMSO-*d*_6_^a^.

Position	δ_C_ mult.	δ_H_ mult. (*J* in Hz)	HMBC Correlation	COSY Correlation	TOCSY Correlation	ROESY Correlation
Hpla 1	173.4, C		Hty-2-NH, Hpla-3a,3b			
2	72.3, CH	4.03, m	Hpla-3a,3b	Hpla-3a,3b		Hpla-3a,3b
2-OH		5.73, brs			Hpla-2,3a,3b	
3	39.4, CH_2_	2.86, dd (14.0,3.6) 2.62, dd (14.0,8.1)	Hpla-5,5′	Hpla-2,3b Hpla-2,3a		
4	128.5, C		Hpla-3a,3b,6,6′			
5,5′	130.6, CH × 2	7.00, d (8.6)	Hpla-3a,3b,5′,5	Hpla-6,6′		Hpla-2,3a,3b
6,6′	114.9, CH × 2	6.63, d (8.6)	Hpla-6′,6	Hpla-5,5′		
7	155.9, C		Hpla-5,5′,6,6′			
Hty 1	169.7, C		Hty-2			
2	50.5, CH	4.49, dt (7.7,5.5)		Hty-3a,3b	Hty-3b,4	Hpla-2, Hty-3a,4, ChoiSul-5b,7ax,7a
2-NH		7.71, d (7.7)		Hty-2,	Hty-2,4	Hpla-2,3a,3b Hty-2,3a,3b, ChoiSul-7ax
3a 3b	34.3, CH_2_	1.77, m 1.69, m	Hty-4	Hty-2,3b,4 Hty-2,3a,4		ChoiSul-7a
4	30.2, CH_2_	2.38, m		Hty-3a,3b		
5	131.6, C		Hty-4,7,7′,			
6,6′	129.1, CH × 2	6.94, d (8.6)	Hty-4,6′,6	Hty-7,7′		Hty-3a,3b,4
7,7′	115.3, CH × 2	6.65, d (8.6)	Hty-7′,7	Hty-6,6′		
8	155.6, C		Hty-6,6′,7,7′			
ChoiSul 1	171.6, C		Hty-2-NH, ChoiSul-2, 3ax			
2	60.1, CH	4.18, dd (9.3,8.8)		ChoiSul-3pe,3pa	ChoiSul-3a,3pe	ChoiSul-3a,3pe, Agm-1-NH
3pe ^b^ 3pa ^b^	30.8, CH_2_	2.02, m 1.77, m	ChoiSul-2,7a	ChoiSul-3a ChoiSul-3a		ChoiSul-3pa ChoiSul-3pe
3a	36.0, CH	2.27, m	ChoiSul-3pa	ChoiSul-3pe,7a,4a,4b		ChoiSul-3pe,4a,4b,7pa
4a 4b	19.6, CH_2_	1.97, m 1.44, m		ChoiSul-3pa,3a,4b,5b ChoiSul-3pa,3a,4a,5b		ChoiSul-4b ChoiSul-4a
5a 5b	23.9, CH_2_	1.76, m 1.38, m		ChoiSul-4a, 4b,5b,6, ChoiSul-4a,4b,5a,6		ChoiSul-5b ChoiSul-5a, Hty-2
6	71.1, CH	4.35, brs		ChoiSul-5a,5b,7pe,7pa	ChoiSul-4a7pe,7pa	ChoiSul-3a,7pe,7pa
7pe ^b^ 7pa ^b^	31.5, CH_2_	2.38, m 1.60, brt (12.0)		ChoiSul-6,7pa,7a ChoiSul-6,7pe,7a		Hty-2,2-NH
7a	54.3, CH	4.03, m		ChoiSul-3a,7pe,7pa	ChoiSul-7pe,7pa,7a	Hty-2,3b ChoiSul-3pa,7pe,7a
Agm 1-NH		7.71, t (5.5)		Agm-1	Agm-1,3	ChoiSul-2, Agm-1,3
1	31.5, CH_2_	3.03, m		Agm-1-NH,2		
2	31.5, CH_2_	1.42, m	Agm-1,3	Agm-1		
3	31.5, CH_2_	1.43, m	Agm-1,2,4			
4	40.4, CH_2_	3.03, m		Agm-3		Agm-3
4-NH		8.58, t (5.6)		Agm-1	Agm-1,3	Agm-1,3
5	157.5, C		Agm-1			
5-NH_2_,NH		7.65, brs				

^a^ 500 MHz for ^1^H, 125 MHz for ^13^C. ^b^ pe: pseudo-equatorial, pa: pseudo-axial.

**Table 3:** NMR Data of Aeruginosin KT575 (**3**) in DMSO-*d*_6_^a^.

Position	δ_C_, Mult.	δ_H_, Mult. (*J* in Hz)	HMBC Correlation	COSY Correlation	TOCSY Correlation	ROESY Correlation
Hpla ^b^ 1	172.3, C		Ile-2,2NH, Hpla-2,2-OH,3a,3b			
2	71.9, CH	4.09, m	Hpla-2-OH,3a,3b	Hpla-2-OH,3a,3b	Hpla-2-OH,3a,3b	Hpla-3a,3b
2-OH		5.98, d (5.8)		Hpla-2	Hpla-2,3a,3b	Ile-2-NH, Hpla-3a, 3b
3a 3b	39.4, CH_2_	2.92, dd (14.0,3.4) 2.70, dd (14.0,7.6)	Hpla-5,9	Hpla-2,3b Hpla-2,3a	Hpla-2,2-OH,3b Hpla-2,2-OH,3a	Hpla-9
4	134.0, C		Hpla-3a,3b,8			
5	130.5, CH	7.22, d (2.1)	Hpla-3a,3pa,8,9	Hpla-9	Hpla-8,9	Hpla-3a,3b
6	123.8, C		Hpla-5,8,9			
7	148.0, C		Hpla-5,8,9			
8	121.3, CH	7.45, d (8.9)		Hpla-9	Hpla-5,9	
9	128.8, CH	7.07, dd (8.9,2.1)	Hpla-3a, 3pb,5	Hpla-5,8	Hpla-5,8	Hpla-3b
Ile 1	168.7, C		Ile-2,2-NH			
2	51.8, CH	4.55, dd (9.4,4.2)	Ile-4a,4b,6	Ile-2-NH,3	Ile-3	Choi-7a
2-NH		7.37, d (9.4)		Ile-2	Ile-2,3,6	Hpla-2-OH, Ile-2
3	38.2, CH	1.52, ddq (4.3,7.1, 6.7)	Ile-2,4a,4b,6	Ile-2,4a,4b,6	Ile-2,4a,4b,5,6	Choi-6-OH
4a 4b	26.3, CH_2_	1.14, m 0.99, m	Ile-5,6	Ile-3,4b,5 Ile-3,4a,5	Ile-3,4b,5,6 Ile-3,4a,5,6	Choi-6-OH
Ile 5	12.0, CH_3_	0.88, t (7.5)	Ile-4a,4b	Ile-4a,4b	Ile-2,3,4a,4b,6	
Ile 6	14.1, CH_3_	0.65, d (6.7)	Ile-2,4a,4b	Ile-3	Ile-3,4a,4b,5	
Choi 1	173.6, C		Choi-1- NH_2_,2,3pe			
1- NH_2_		7.29, d (1.0) 6.83, d (1.0)		Choi-1- NHb Choi-1- NHa	Choi-1- NHb Choi-1- NHa	Choi-2 Choi-2
2	59.7, CH	4.14, dd (9.6,8.4)	Choi-1- NHb,3pe,7a	Choi-3pa,3pe	Choi-3pa, 3pe,3a,4pa	Choi-3pa,3pe
3pe ^c^ 3pa ^c^	30.6, CH_2_	1.97, m 1.79, ddq (12.5,9.8,7.8)	Choi-2,3pe,7a	Choi-2,3pe, 3a Choi-2,3pa,3a	Choi-2,3pe, 3a Choi-2,3pa,3a	
3a	36.3, CH	2.24, ddq (12.9,7.1,6.0)	Choi-2,3pe, 3pa,4pa	Choi-3pe,3pa, 4pe,4pa,7a	Choi-2,3pe, 3pa,4pa,6,7pe, 7pa	
4pe ^c^ 4pa ^c^	19.2, CH_2_	2.02, m 1.41, m		Choi-3a,4pa Choi-3a,4pe	Choi-3pe,3pa,3a,4pa Choi-3pe,3pa,3a,4pe	
5	26.2, CH_2_	1.42, m	Choi-3a,7pa	Choi-4pe,6		
6	64.1, CH	3.90, brs	Choi-4pa,6-OH,7pe	Choi-4pa,6-OH,7pe,7pa	Choi-3pe,3pa, 3a,4pe,4pa,7a	Choi-4pa, 6-OH,7pe
6-OH		4.53, d (3.1)		Choi-6	Choi-4pe,4pa, 5,6,7pe,7pa	Choi-5,6, 7pa, Ile-3,4a
7pe ^c^ 7pa ^c^	33.7, CH_2_	1.96, m 1.69, brtd (11.8, 1.9)	Choi-6-OH	Choi-3a,7pa Choi-3a,7pe	Choi-2,3pe, 3a,4pa,6,7a,7pa Choi-2,3a,5, 6,7a,7pa	
7a	54.0, CH	4.07, m	Choi-4pa, 7pe,7pa	Choi-7pe,7pa	Choi-3a,3pe, 3pa,6,7pe,7pa	Ile-2, Choi-3a,4pa

^a^ 400 MHz for ^1^H and 100 MHz for ^13^C. ^b^ 6-Cl-Hpla-7-sulfate. ^c^ pe: pseudo-equatorial, pa: pseudo-axial.

## Data Availability

Data are contained within the article and Supplementary Materials.

## Supplementary Materials

The following supporting information can be downloaded at https://www.mdpi.com/article/10.3390/md22090389/s1, 1D ^1^H, ^13^C and 2D (HSQC, HMBC, COSY, TOCSY, ROESY) NMR spectra, HRMS, MS/MS data, and full NMR tables of the new compounds (**1**–**3**).

## References

[1] Chlipala G.E., Mo S., Orjala J. (2011). Chemodiversity in freshwater and terrestrial cyanobacteria—A source for drug discovery. Curr. Drug Targets.

[2] Shah S.A.A., Akhter N., Auckloo B.N., Khan I., Lu Y., Wang K., Wu B., Guo Y.-W. (2017). Structural diversity, biological properties and application of natural products from cyanobacteria. A review. Mar. Drugs.

[3] Huang I.S., Zimba P.V. (2019). Cyanobacterial bioactive metabolites—A review of their chemistry and biology. Harmful Algae.

[4] Bishoyi A.K., Sahoo C.R., Padhy R.N. (2023). Recent progression of cyanobacteria and their pharmaceutical utility: An update. J. Biomol. Struct. Dyn..

[5] Taori K., Liu Y., Paul V.J., Luesch H. (2009). Combinatorial strategies by marine cyanobacteria: Symplostatin 4, an antimitotic natural dolastatin 10/15 hybrid that synergizes with the coproduced HDAC inhibitor largazole. ChemBioChem.

[6] Carmeli S., Moore R.E., Patterson M.L. (1991). Mirabimides A-D, new N-acylpyrrollinones from the blue-green alga *Scytonema mirabille*. Tetrahedron.

[7] Linington R.G., González J., Ureña L.D., Romero L.I., Ortega-Barría E., Gerwick W.H. (2007). Venturamides A and B: Antimalarial constituents of the Panamanian marine cyanobacterium *Oscillatoria* sp.. J. Nat. Prod..

[8] Ziemert N., Ishida K., Quillardet P., Bouchier C., Hertweck C., de Marsac N.T., Dittmann E. (2008). Microcyclamide biosynthesis in two strains of Microcystis aeruginosa: From structure to genes and vice versa. Appl. Environ. Microbiol..

[9] An T., Kumar T.K.S., Wang M., Liu L., Lay J.O., Liyanage R., Berry J., Gantar M., Gawley R.E., Rein K. (2007). Structure of pahayoklides A and B, cyclic peptides from a *Lyngby* sp.. J. Nat. Prod..

[10] Pargament I., Carmeli S. (1994). Schizotrin A, a novel antimicrobial cyclic peptide from a cyanobacterium. Tetrahedron Lett..

[11] Taori K., Matthew S., Rocca J.R., Paul V.J., Luesch H. (2007). Lyngbyastatins 5–7, potent elastase inhibitors from Floridian marine cyanobacteria, *Lyngbya* spp.. J. Nat. Prod..

[12] Murakami M., Kodani S., Ishida K., Matsuda H., Yamaguchi K. (1997). Micropeptin 103, a chymotrypsin inhibitor from the cyanobacterium *Microcystis viridis* (NIES-103). Tetrahedron Lett..

[13] Schmidt E.W., Harper M.K., Faulkner D.J. (1977). Mozamides A and B, cyclic peptides from a theonelid sponge from Mozambique. J. Nat. Prod..

[14] Harad K.-i., Fujii K., Shimada T., Suzuki M. (1995). Two cyclic peptides, anabaenopeptines, a third group of bioactive compounds from the cyanobacterium *Anabaena flos-aquae* NRC 525-17. Tetrahedron Lett..

[15] Carroll A.R., Pierens G.K., Fechner G., De Almeida L.P., Ngo A., Simpson M., Hyde E., Hooper J.N., Boström S.L., Musil D. (2002). Dysinosin A: A novel inhibitor of factor VIIa and thrombin from a new genus and species of Australian sponge of the family dysideidae. J. Am. Chem. Soc..

[16] Murakami M., Okita Y., Ishida K., Matsuda H., Yamaguchi K. (1994). Aeruginosin 298-A, a thrombin and trypsin inhibitor from the blue-green alga *Microcystis aeruginosa* (NIES-298). Tetrahedron Lett..

[17] Ersmark K., Del V.J.R., Hanessian S. (2008). Chemistry and biology of the aeruginosin family of serine protease inhibitors. Angew. Chem. Int. Ed..

[18] Liu J., Zhang M., Huang Z., Fang J., Wang Z., Zhou C., Qiu X. (2023). Diversity, Biosynthesis and Bioactivity of Aeruginosins, a Family of Cyanobacteria-Derived Nonribosomal Linear Tetrapeptides. Mar. Drugs.

[19] Lifshitz M., Carmeli S. (2012). Metabolites of a *Microcystis aeruginosa* bloom material from Lake Kinneret, Israel. J. Nat. Prod..

[20] Scherer M., Gademann K. (2017). Total Synthesis and Structural Revision of Aeruginosin KT608A. Org. Lett..

[21] Ninio S., Lupu A., Viner-Mozzini Y., Zohary T., Sukenik A. (2020). Multiannual variations in Microcystis bloom episodes—Temperature drives shift in species composition. Harmful Algae.

[22] Kaplan-Levy R.N., Alster-Gloukhovski A., Benyamini Y., Zohary T. (2016). Lake Kinneret phytoplankton: Integrating classical and molecular taxonomy. Hydrobiologia.

[23] Weisthal Algor S., Sukenik A., Carmeli S. (2023). Hydantoanabaenopeptins from Lake Kinneret *Microcystis* bloom, Isolation, and Structure Elucidation of the Possible Intermediates in the Anabaenopeptins Biosynthesis. Mar. Drugs.

[24] Elkobi-Peer S., Faigenbaum R., Carmeli S. (2012). Bromine- and Chlorine-Containing Aeruginosins from *Microcystis aeruginosa* Bloom Material Collected in Kibbutz Geva, Israel. J. Nat. Prod..

[25] Marfey P. (1984). Marfey’s reagent: 1-fluoro-2,4-dinitrophenyl-5-L-alanine amide. Carlsberg Res. Commun..

[26] Fujii K., Sivonen K., Adachi K., Noguchi K., Shimizu Y., Sano H., Hirayama K., Suzuki M., Harada K. (1997). Comparative Study of Toxic and Non-toxic Cyanobacterial Products: A Novel Glycoside, Suomilide, from Non-toxic *Nudularia spumigena* HKVV. Tetrahedron Lett..

[27] Ishida K., Okita Y., Matsuda H., Okino T., Murakami M. (1999). Aeruginosins, protease inhibitors from the cyanobacterium *Microcystis aeruginosa*. Tetrahedron.

[28] Adiv S., Carmeli S. (2013). Protease inhibitors from a *Microcystis aeruginosa* bloom material collected from the Dalton water reservoir, Israel. J. Nat. Prod..

[29] Hasin O., Carmeli S. (2018). Isolation and elucidation of structures of secondary metabolites from a *Microcystis* sp. Bloom material collected in southern Israel. Nat. Prod. Commun..

[30] Fujii K., Ikai Y., Mayumi T., Oka H., Suzuki M., Harada K.-I. (1997). A Nonempirical Method Using LC/MS for Determination of the Absolute Configuration of Constituent Amino Acids in a Peptide: Elucidation of Limitations of Marfey’s Method and of Its Separation Mechanism. Anal. Chem..

[31] Sukenik A., Rosin C., Porat R., Teltsch B., Banker R., Carmeli S. (1998). Toxins from Cyanobacteria and Their Potential Impact on Water Quality of Lake Kinneret, Israel. Isr. J. Plant Sci..

[32] Beresovsky D., Hadas O., Livne A., Sukenik A., Kaplan A., Carmeli S. (2006). Toxins and Biologically Active Secondary Metabolites of Microcystis sp. isolated from Lake Kinneret. Isr. J. Chem..

