# Supplementary material for: Sulfated Aeruginosins from Lake Kinneret: Microcystis Bloom, Isolation, Structure Elucidation, and Biological Activity
Source: Mar Drugs. 2024 Aug 28;22(9):389. doi: 10.3390/md22090389 (PMC11433283; doi:10.3390/md22090389)
Supplement: Supplementary file 1 [file marinedrugs-22-00389-s001.zip › marinedrugs-3172066-supplementary.pdf]

Sulfated Aeruginosins from Lake Kinneret *Microcystis* bloom, Isolation, Structure Elucidation and Biological Activity

Shira Weisthal Algor,[1] Assaf Sukenik[2] and Shmuel Carmeli.[1*]

[1]Raymond and Beverly Sackler Faculty of Exact Sciences, School of Chemistry, Tel Aviv University, Tel Aviv 69978, Israel; shiraw1@mail.tau.ac.il
[2]The Yigal Allon Kinneret Limnological Laboratory, Israel Oceanographic & Limnological Research Institute, P.O. Box 447, Migdal 49500, Israel; assaf@ocean.org.il
[*]Correspondence: carmeli@tauex.tau.ac.il; Tel.: +972-54-3117290

Supplementary Material

Table of Content

Page	Title
S2-S3	Table of Content
S4-S11	Table S1**.** 86 isolated and fully characterized (5 from sponge) aeruginosins + 46 characterized by LCMSMS
S12	Figure S1. ^{1}H NMR Spectrum of Aeruginosin KT688 (**1**) in DMSO-d_6
S13	Figure S2. ^{13}C NMR Spectrum of Aeruginosin KT688 (**1**) in DMSO-d_6
S14	Figure S3. HSQC Spectrum of Aeruginosin KT688 (**1**) in DMSO-d_6
S15	Figure S4. HMBC Spectrum of Aeruginosin KT688 (**1**) in DMSO-d_6
S16	Figure S5. COSY Spectrum of Aeruginosin KT688 (**1**) in DMSO-d_6
S17	Figure S6. TOCSY Spectrum of Aeruginosin KT688 (**1**) in DMSO-d_6
S18	Figure S7. ROESY Spectrum of Aeruginosin KT688 (**1**) in DMSO-d_6
S19-S20	Table S2. NMR Data of the Minor *cis* Rotamer of Aeruginosin KT688 (**1**) in DMSO-d_6[a]
S21	Figure S8. Positive HR ESI MS/MS Spectrum of Aeruginosin KT688 (**1**)
S22	Table S3. Comparison of the chemical shifts of the Choi-6-sulfate moiety of the *trans* rotamer of Aeruginosins KT688 (**1**) and GE766
S23-S25	Table S4. NMR Data of the Major *cis* Rotamer of Aeruginosins KT608A, KT608B and Minor *cis* KT688 (**1**) in DMSO-d_6[a]
S26	Figure S9. LCMS Traces of Marfey's Method Amino Acids Analysis of Aeruginosin KT688 (**1**)
S27	Figure S10. HPLC Traces of Chiral Chromatography of HPLA Analysis of Aeruginosin KT688 (**1**)
S28	Figure S11. ^{1}H NMR Spectrum of Aeruginosin KT718 (**2**) in DMSO-d_6
S29	Figure S12. ^{13}C NMR Spectrum of Aeruginosin KT718 (**2**) in DMSO-d_6
S30	Figure S13. HSQC Spectrum of Aeruginosin KT718 (**2**) in DMSO-d_6
S31	Figure S14. HMBC Spectrum of Aeruginosin KT718 (**2**) in DMSO-d_6
S32	Figure S15. COSY Spectrum of Aeruginosin KT718 (**2**) in DMSO-d_6
S33	Figure S16. TOCSY Spectrum of Aeruginosin KT718 (**2**) in DMSO-d_6
S34	Figure S17. ROESY Spectrum of Aeruginosin KT718 (**2**) in DMSO-d_6
S35-S36	Table S5. NMR Data of the Minor *cis* Rotamer of Aeruginosin KT718 (**2**) in DMSO-d_6[a]
S37	Figure S18. Positive HR ESI MS/MS Spectrum of Aeruginosin KT718 (**2**)
S38	Figure S19. LCMS Traces of Marfey's Method Amino Acids Analysis of Aeruginosin KT718 (**2**)
S39	Figure S20. HPLC Traces of Chiral Chromatography of HPLA Analysis of Aeruginosin KT718 (**2**)
S40	Figure S21. ^{1}H NMR Spectrum of Aeruginosin KT575 (**3**) in DMSO-d_6
S41	Figure S22. ^{13}C NMR Spectrum of Aeruginosin KT575 (**3**) in DMSO-d_6

S42	Figure S23. HSQC Spectrum of Aeruginosin KT575 (**3**) in DMSO-d_6
S43	Figure S24. HMBC Spectrum of Aeruginosin KT575 (**3**) in DMSO-d_6
S44	Figure S25. COSY Spectrum of Aeruginosin KT575 (**3**) in DMSO-d_6
S45	Figure S26. TOCSY Spectrum of Aeruginosin KT575 (**3**) in DMSO-d_6
S46	Figure S27. ROESY Spectrum of Aeruginosin KT575 (**3**) in DMSO-d_6
S47	Figure S28. Negative HR ESI MS/MS Spectrum of Aeruginosin KT575 (**3**)
S48	Figure S29. LCMS Traces of Marfey's Method Amino Acids Analysis of Aeruginosin KT575 (**3**)
S49	Table S6. Comparison of the ^{1}H and ^{13}C NMR data of the 6-Cl-Hpla-7-sulfate moiety in Aeruginosin KT575 (**3**) and Aeruginosin 89-B in DMSO-d_6
S50	Table S7. Comparison of the ^{1}H and ^{13}C NMR data of the Choi moiety of Aeruginosin KT575 (**3**) and Aeruginosin DA495B in DMSO-d_6[a]

Table S1. 86 isolated and fully characterized (5 from sponge) aeruginosins + 46 characterized by LCMSMS.

Cyanobacterium	Name/MW	Residue 1[a]	Residue 2	Residue 3	Residue 4	Activity (μM)
M. aeruginosa NIES-89[1]	Aeruginosin 89-A 717/719, MH^+	*m*-Cl-D-Hpla-O-sulfate	D-Leu	L-Choi	L-Argal	Trypsin (0.56) Thrombin (0.04)
M. aeruginosa NIES-89[1]	Aeruginosin 89-B 717/719, MH^+	*m*-Cl-D-Hpla-O-sulfate	D-Leu	L-Choi	D-Argal	Trypsin (9.2) Thrombin (0.07)
M. aeruginosa NIES-98[2]	Aeruginosin 98-A 689/691, MH^+	*m*-Cl-D-Hpla	D-*allo*Ile	L-Choi-6-*O*-sulfate	Agm	Trypsin (0.87) Thrombin (10.1)
M. aeruginosa NIES-98[2]	Aeruginosin 98-B 655, MH^+	D-Hpla	D-*allo*Ile	L-Choi-6-*O*-sulfate	Agm	Trypsin (0.92) Thrombin (15.3)
M. aeruginosa NIES-98[1]	Aeruginosin 98-C 731/733, MH^+	*m*-Br-D-Hpla	D-*allo*Ile	L-Choi-6-*O*-sulfate	Agm	Trypsin (5.3) Thrombin (4.1)
M. aeruginosa NIES-101[1]	Aeruginosin 101 721/723/725, MH^+	*m,m*-di-Cl-D-Hpla	D-*allo*Ile	L-Choi-6-*O*-sulfate	Agm	Trypsin (4.1) Thrombin (4.4)
M. viridis NIES-102[3]	Aeruginosin 102-A 733, MH^+	D-Hpla-O-sulfate	D-Tyr	L-Choi	L-Argal	Trypsin (0.27) Thrombin (0.05)
M. viridis NIES-102[3]	Aeruginosin 102-B 733, MH^+	D-Hpla-O-sulfate	D-Tyr	L-Choi	D-Argal	Trypsin (1.5) Thrombin (0.14)
M. viridis NIES-103[4]	Aeruginosin 103-A 681, MH^+	D-Hpla	D-Tyr	L-Choi	L-Argal-O-ethyl	Trypsin (75.0) Thrombin (13.2)
O. agardhii NIES-205[5]	Aeruginosin 205-A 807/805, MH^+	D-Hpla-2-O-sulfate// D-Plac	D-Xyl-3-O-Leu?// 3-Cl-Leu	L-6-Cl-Choi?// D-4'-sulfate-Xyl-6-O-L-Choi	Agm	Trypsin (0.09) Thrombin (1.86)
O. agardhii NIES-205[5]	Aeruginosin 205-B 807/805, MH^+	L-Hpla-2-O-sulfate// L-Plac	D-Xyl-3-O-Leu?// 3-Cl-Leu	L-6-Cl-Choi?// D-4'-sulfate-Xyl-6-O-L-Choi	Agm	Trypsin (0.09) Thrombin (0.21)
M. aeruginosa NIES-298[6,7]	Aeruginosin 298-A	D-Hpla	D-Leu	L-Choi	L-Argol	Trypsin (1.6) Thrombin (0.49)
M. aeruginosa NIES-298[1,8]	Aeruginosin 298-B 462, MH^+	D-Hpla	D-Leu	L-Choi-amide	-	Trypsin (>200) Thrombin (>200)
Microcystis sp.[9]	Microcin SF608	L-Hpla	L-Phe	L-Choi	Agm	Trypsin (0.82)

M. aeruginosa[10,11]	Aeruginosin EI461	L-Hpla	D-Leu	L-di*epi*Choi-amide	-	Trypsin (>100) Thrombin (?)
O. agardhii[12,13]	Oscillarin	D-Plac	D-Phe	L-Choi	Adc	Trypsin (0.56) Thrombin (0.04)
Planktothrix agardhii CYA126[14]	Aeruginoside 126A	D-Plac	D-Leu	Xyl-6-O-L-Choi	Adc	Trypsin (0.56) Thrombin (0.04)
Planktothrix agardhii CYA126[14]	Aeruginoside 126A	D-Plac	D-Leu	Xyl-6-O-L-Choi	Agm	Trypsin (0.56) Thrombin (0.04)
Microcystis sp. IL-323[15]	Aeruginosin KY642	*m,m*-di-Cl-L-Hpla	D-*allo*Ile	L-Choi	Agm	Trypsin (2.1) Thrombin (?)
Microcystis sp. IL-323[15]	Aeruginosin KY608	*m*-Cl-L-Hpla	D-*allo*Ile	L-Choi	Agm	Trypsin (2.8) Thrombin (?)
M. aeruginosa IL-337[16]	Aeruginosin GH553	L-Hpla	D-Tyr	D-di*epi*Choi-amide	-	Trypsin (100) Thrombin (>100)
M. aeruginosa IL-347[16]	Aeruginosin KT608A	L-Hpla	D-Phe	D-di*epi*Choi	Agm	Trypsin (1.9) Thrombin (enh)
M. aeruginosa IL-347[16]	Aeruginosin KT608B	D-Hpla	D-Phe	D-di*epi*Choi	Agm	Trypsin (1.3) Thrombin (100)
M. aeruginosa IL-347[16]	Aeruginosin KT650	D-Hpla	D-Phe	D-di*epi*Choi	Agm	Trypsin (19.9) Thrombin (>100)
M. aeruginosa IL-347[16]	Pseudoaeruginosin KT554	L-Hpla	D-Leu	L-Phe	Agm	Trypsin (100) Thrombin (>100)
M. aeruginosa PCC 7806[17]	Aeruginosin 686A	*m*-Cl-L-Hpla	D-Tyr	Choi	Argal	Trypsin (0.56) Thrombin (0.04)
M. aeruginosa PCC 7806[17]	Aeruginosin 686B	*m*-Cl-L-Hpla	D-Tyr	Choi	Argal	Trypsin (0.56) Thrombin (0.04)
Lamellodysidea chlorea[18]	Dysinosin A	D-Mgs	D-Leu	5-OH-L-Choi	Adc	Trypsin (0.56) Thrombin (0.04)

Lamellodysidea chlorea[19,20]	Chlorodysinosin A	D-Mgs	(3*R*)-Cl-D-Leu	5-OH-L-Choi	Adc	Trypsin (0.56) Thrombin (0.04)
Lamellodysidea chlorea[21]	Dysinosin B	D-Mgs	Val	6-Glu-5-OH-L-Choi	Adc	Trypsin (0.56) Thrombin (0.04)
Lamellodysidea chlorea[21]	Dysinosin C	D-Mgs	Val	5-OH-L-Choi	Adc	Trypsin (0.56) Thrombin (0.04)
Lamellodysidea chlorea[21]	Dysinosin D	D-Mgs	Val	5-OH-L-Choi	Adc	Trypsin (0.56) Thrombin (0.04)
Nodularia (sphaerocarpa) spumigena AV1[22]	Suomilide	D-Mgs	*allo*-Ile	2,6-di-HA-□-Glu-7-O-Abn	Adc	Trypsin (0.56) Thrombin (0.04)
Nostoc sp. IL-235[23]	Banyaside A	Mgs	D-Leu	2-HA-3-carbamyl-α-Glu-7-O-Abn	Adc	Trypsin (0.56) Thrombin (0.04)
Nostoc sp. IL-235[23]	Banyaside B	Mgs	D-Leu	6-HA-α-Glu-7-O-Abn	Adc	Trypsin (0.56) Thrombin (0.04)
M. aeruginosa IL-374[24]	Aeruginosin DA495A	D-Hpla	D-Phe	L-6-*epi*Choi amide	-	Trypsin (>100) Thrombin (>100)
M. aeruginosa IL-374[24]	Aeruginosin DA511	D-Hpla	D-Tyr	L-6-*epi*Choi amide	-	Trypsin (>100) Thrombin (>100)
M. aeruginosa IL-374[24]	Aeruginosin DA642A	*m*-Cl-L-Hpla	L-Phe	L-Choi	Agm	Trypsin (30.8) Thrombin (15.5)
M. aeruginosa IL-374[24]	Aeruginosin DA642B	*m*-Cl-D-Hpla	L-Phe	L-Choi	Agm	Trypsin (19.0) Thrombin (>100)
M. aeruginosa IL-374[24]	Aeruginosin DA688	*m*-Cl-D-Hpla	D-Leu	L-Choi-6-sulfate	Agm	Trypsin (9.5) Thrombin (>100)
M. aeruginosa IL-374[24]	Aeruginosin DA722	*m,m*-di-Cl-D-Hpla	D-Leu	L-Choi-6-sulfate	Agm	Trypsin (7.3) Thrombin (>100)
M. aeruginosa IL-374[24]	Aeruginosin DA495B	*m*-Cl-D-Hpla	D-Leu	L-Choi amide	-	Trypsin (>100) Thrombin (>100)

M. aeruginosa IL-377[25]	Aeruginosin GE642	*m,m*-di-Cl-D-Hpla	D-Leu	L-Choi	Agm	Trypsin (8.5) Thrombin (>100)
M. aeruginosa IL-377[25]	Aeruginosin GE686	*m,m*-Br,Cl-D-Hpla	D-*allo*Ile	L-Choi	Agm	Trypsin (3.2) Thrombin (12.8)
M. aeruginosa IL-377[25]	Aeruginosin GE766	*m,m*-Br,Cl-D-Hpla	D-*allo*Ile	L-Choi-6-*O*-sulfate	Agm	Trypsin (12.2) Thrombin (>100)
M. aeruginosa IL-377[25]	Aeruginosin GE730	*m,m*-di-Br-D-Hpla	D-*allo*Ile	L-Choi	Agm	Trypsin (2.3) Thrombin (12.9)
M. aeruginosa IL-377[25]	Aeruginosin GE810	*m,m*-di-Br-D-Hpla	D-*allo*Ile	L-Choi-6-*O*-sulfate	Agm	Trypsin (18.2) Thrombin (>100)
M. IN-1[26]	Aeruginosin IN608	*m*-Cl-D-Hpla	D-Leu	L-Choi	Agm	Trypsin (4.3) Thrombin (?)
M. IN-1[26]	Aeruginosin IN652	*m*-Br-D-Hpla	D-Leu	L-Choi	Agm	Trypsin (4.1) Thrombin (?)
N. spumigena[27]	Spumigin A	D-Hpla	D-Hty	L-(4S)-MePro	Argol	
N. spumigena[27]	Spumigin B1/B2	D-Hpla	D-Hty	L-(4S)-MePro	Arg	
N. spumigena[27]	Spumigin C	D-Hpla	D-Hty	L-Pro	Arg	
N. spumigena[27]	Spumigin D	D-Hpla	D-Hty	L-Pro	Argol	
N. spumigena[27]	Spumigin E	D-Hpla	D-Hty	L-(4S)-MePro	Argal	
N. spumigena[27]	Spumigin F	D-Hpla	D-Hty	L-Pro	Argal	
N. spumigena[27]	Spumigin G	D-Hpla	D-Hph	L-(4S)-MePro	Argal	
N. spumigena[27]	Spumigin H	D-Hpla	D-Hph	L-Pro	Argal	
N. spumigena[28]	Aeruginosin NAL1	Bu	Tyr	Choi	Argal	
N. spumigena[28]	Aeruginosin NAL2	Hex	Tyr	Choi	Argal	
N. spumigena[28]	Aeruginosin NAL3	Oct	Tyr	Choi	Argal	
N. spumigena[28]	Aeruginosin NAL4	Oct	Tyr	Choi-pentose	Argal	

N. spumigena[28]	Aeruginosin NOL1	Ac	Tyr	Choi	Argol	
N. spumigena[28]	Aeruginosin NOL2	Bu	Tyr	Choi	Argol	
N. spumigena[28]	Aeruginosin NOL3	Hex	Tyr	Choi	Argol	
N. spumigena[28]	Aeruginosin NOL4	Oct	Tyr	Choi	Argol	
N. spumigena[28]	Aeruginosin NOL5	Hex	Tyr	Choi-pentose	Argol	
N. spumigena[28]	Aeruginosin NOL6	Oct	Tyr	Choi-pentose	Argol	
N. spumigena[28]	Aeruginosin NOL7	Dec	Tyr	Choi-pentose	Argol	
Microcystis sp.[29]	Aeruginosin KB676	D-Hpla	D-Phe	L-6-*epi*-Choi	^{4}*N*-prenyl-agmatine	Trypsin (40.0)
Microcystis sp.[30]	Aeruginosin LH650A	D-Cl-Hpla	D-Leu	L-Choi	L-Amap	Trypsin (37.9) Thrombin (1.8)
Microcystis sp.[30]	Aeruginosin LH650B	D-Cl-Hpla	D-Leu	L-Choi	L-Amap	Trypsin (35.3) Thrombin (1.8)
Microcystis sp.[30]	Aeruginosin LH606	D-Cl-Hpla	D-Leu	L-Choi	Aap	Trypsin (18.5) Thrombin (2.5)
Nodularia spumigena[31]	Pseudoaeruginosin NS1	Hexanoate	L-Tyr	L-(4S)-MePro	Argal	Trypsin (0.19)
Nodularia spumigena[31]	Pseudoaeruginosin NS2	Hexanoate	L-Tyr	L-(4S)-MePro	Argol	
Nostoc sp. CENA543[32]	Pseudospumigin A 613	Hpla	D-Hty	L-Ile	Argininal	Trypsin (4.5)
Nostoc sp. CENA543[32]	Pseudospumigin B 613	Hpla	Hty	Leu	Argininal	

Nostoc sp. CENA543[32]	Pseudospumigin C 599	Hpla	Hty	Val	Arginal	
Nostoc sp. CENA543[32]	Pseudospumigin D 597	Hpla	Hph	Ile	Arginal	
Nostoc sp. CENA543[32]	Pseudospumigin E 597	Hpla	Hph	Leu	Arginal	
Nostoc sp. CENA543[32]	Pseudospumigin F 583	Hpla	Hph	Val	Arginal	
Microcystis sp.[33]	Aeruginosin TR642	D-Hpla	D-*allo*Ile	(2R,3aR,6R,7aR)-Choi	4,5-didehydroaraginal	Trypsin (3.8) Thrombin (0.85)
M. sp.[34]	Aeruginosin BH462A	L-Hpla	D-*allo*Ile	L-Choi	-	Tryp -
M. sp.[34]	Aeruginosin BH462B	L-Hpla	D-Leu	L-Choi	-	Tryp -
M. sp.[34]	Aeruginosin BH604	L-Hpla	L-*allo*Ile	L-Choi	L-Argol	Tryp -
Nostoc sp. UHCC 0870[35]	Varlaxin 1046A	Mgs	D-Ile	L-Choi-a-D-Glu(Hpaa)$_2$	Aaep	Tryp (0.62-3.6 nM)
Nostoc sp. UHCC 0870[35]	Varlaxin 1022A	Mgs	D-Ile	L-Choi-a-D-Glu(Hpaa)$_2$	Agm	Tryp (97-230 nM)
Nostoc spp.[35]	+22 variants by LCMSMS					
Nodularia spumigena[36]	9 spumigins + 4 aeruginosins by LCMSMS			Pro/MePro/Choi		
Bloom material[37]	11 variants by LCMSMS	Hpla/Pla		5-OH-Choi/Choi-glyco-FA(4-8)	Agm	

[a]Order of the residues from the N-terminus to the C-terminus. Hpla – *p*-hydroxyphenyllactic acid; *m*-Cl-Hpla – *m*-chloro-*p*-hydroxyphenyllactic; *m,m*-di-Cl-L-Hpla – *m,m*-dichloro-*p*-hydroxyphenyllactic; *m*-Br-D-Hpla – *m*-bromo-*p*-hydroxyphenyllactic; Plac – phenyllactic acid; L-Choi – (2S,3aS,6R,7aS)-2-carboxy-6-hydroxyoctahydroindole; L-di*epi*Choi – (2S,3aR,6R,7aR)-2-carboxy-6-hydroxyoctahydroindole; D-di*epi*Choi –

(2R,3aR,6R,7aR)-2-carboxy-6-hydroxyoctahydroindole; Agm – agmatine; Argal – dihydroarginine; Argol – tetrahydroarginine; Adc – 1-(*N*-amidino-Δ^3-pyrrolino)ethyl.

References

1. Ishida, K.; Okita, Y.; Matsuda, H.; Okino, T.; Murakami, M., *Tetrahedron*, **1999**, *55*, 10971-10988.
2. Murakami, M.; Ishida, K.; Okino, T.; Okita, Y.; Matsuda, H.; Yamaguchi, K., *Tetrahedron Lett.* **1995**, *36*, 2785-2788.
3. Matsuda, H.; Okino, T.; Murakami, M.; Yamaguchi, K., *Tetrahedron*, **1996**, *52*, 14501-14506.
4. Kodani, S.; Ishida, K.; Murakami, M., *J. Nat. Prod.*, **1998**, *61*, 1046-1048.
5. Shin, H. J.; Matsuda, H.; Murakami, M.; Yamaguchi, K., *J. Org. Chem.*, **1997**, *62*, 1810-1813.
6. Murakami, M.; Okita, Y.; Matsuda, H.; Okino, T.; Yamaguchi, K., *Tetrahedron Lett.* **1994**, *35*, 3129-3132.
7. Valls, N.; Lopez-Canet, M; Vallribera, M.; Bonjoch, J. *J. Am. Chem. Soc.* **2000**, *122*, 11248-11249.
8. Valls, N.; Lopez-Canet, M; Vallribera, M.; Bonjoch, J. *Chem. Eur. J.* **2001**, *7*, 3446-3460.
9. Banker, R.; Carmeli, S., *Tetrahedron*, **1999**, *55*, 10835-10844.
10. Ploutno, A.; Shoshan, M.; Carmeli, S., *J. Nat. Prod.* **2002**, *65*, 973-978.
11. Valls, N.; Vallribera, M.; Carmeli, S.; Bonjoch, J. *Org. Lett*. **2003**, *5*, 447-450.
12. Konetschny-Rapp, S.; Krell, H.-W.; Martin, U.' *PCT WO96/11941*, **1996**.
13. Hanessian, S.; Tremblay, M.; Petersen, J. F. W. *J. Am. Chem. Soc.* **2004**, *126*, 6064-6071.
14. Ishida, K.; Christiansen, G.; Yoshida, W. Y.; Kurmayer, R.; Welker, M.; Valls, N.; Bonjoch, J.; Hertweck, C.; Borner, T.; Hemscheidt, T.; Dittmann, E., *Chem. Biol.*, **2007**, *14*, 565-576.
15. Raveh, A.; Carmeli, S., *Phytochem. Lett.*, **2009**, *2*, 10-14.
16. Lifshits, M.; Carmeli, S. *J. Nat. Prod.* **2012**, *75*, 209-219.
17. Ishida, K.; Welker, M.; Christiansen, G.; Cadel-Six, S.; Couchier, C.; Dittmann, E.; Hertweck,, C.; Tanddeau de Marsac, N. *Appl. Environ. Microbiol.* **2009**, *75*, 2017-2029.
18. Carroll, A. R.; Pierens, G. K.; Fechner, G.; de Almeida Leone, P.; Ngo, A.; Simpson, M.; Hyde, E.; Hooper, J. N. A.; Bostrom, S.-L.; Musil, D.; Quinn, R. J.; *J. Am. Chem. Soc.* **2002**, *124*, 13340-13341.
19. Goetz, G. H.; Harrigan, G. G.; Likos, J. J.; Kasten, T. P. *PCT WO03/051831*, **2003**.
20. Hanessian, S.; Del Valle, J. R.; Xue, Y.; Blomberg, N. *J. Am. Chem. Soc.* **2006**, *128*, 10491-10495.
21. Carroll, A. R.; Buchanan, M. S.; Edser, A.; Hyde, E.; Simpson, M.; Quinn, R. J. *J. Nat. Prod.* **2004**, *67*, 1291-1294.
22. Fujii, K.; Sivonen, K.; Adachi, K.; Noguchi, K.; Shimizu, Y.; Sano, H.; Hirayama, K.; Suzuki, M.; Harada, K.-i. *Tet Lett*. **1997***, 38*, 5529-5532.
23. Pluotno, A.; Carmeli, S. *Tetrahedron* **2005**, *61*, 575-583.

24. Adiv, S.; Carmeli, S. *J. Nat. Prod*. **2013**, *76*, 2307-2315.
25. Alkobi-Peer, S.; Faigenbaum, R.; Carmeli, S. *J. Nat. Prod*. **2012**, *75*, 2144-2151.
26. Alkobi-Peer, S.; Singh, R. K.; Mohapatra, T. M.; Tiwari, S. P.; Carmeli, S. *J. Nat. Prod*. **2013**, *76*, 1187-1190.
27. Fujii, K., Sivonen, K., Adachi, K., Noguchi, K., Sano, H., Hirayama, K., et al. *Tetrahedron Lett* **1997**, *31*, 5525–5528.
28. Fewer, D. P.; Jokela, J.; Paukku, E.; Osterholm, J.; Wahlsten, M.; Permi, P.; Aitio, O.; Rouhianen, L.; Gomez-Saez, G. V.; Sivonen, K. *PLoS ONE*, **2013**, *8*, e73618.
29. Elkobi-Peer, S.; Carmeli, S. *Mar. Drugs* **2015**, *13*, 2347-2375.
30. Vegman, R.; Carmeli, S. *Tetrahedron*, **2014**, *70*, 6817-6824.
31. Liu, L. Budnjo, A.; Jokela, J.; Haug, B. E.; Fewer, D. P.; Wahlsten, M.; Rouhiainen, L.; Permi, P.; Fossen, T.; Sivonen, K. *ACS Chem. Biol.* **2015**, *10*, 725-733.
32. Jokela, J.; Heinila, L. M.; Shishido, T. K.; Wahlsten, M.; Fewer, D. P.; Fiore, M. F.; Wang, H.; Haapaniemi, E.; Permi, P.; Sivonen, K. *Front. Microbiol*. **2017**, 1963; doi: 10.3389/fmicb.2017.01963.
33. Hassan-Amer, R.; Carmeli, S. *Mar. Drugs* **2017**, *15,* 371.
34. Hasin, H.; Carmeli, S. *Nat. Prod. Comm.* **2018**, *13*, 1313-1318.
35. Heinila, L.M.P.; Jokela, J.; Ahmed, M.N.; Wahlsten, M.; Kumar, S.; Hrouzek, P.; Permi, P.; Koistenen, H.; Fewer, D.P.; Sivonen, K. Org. Biomol. Chem. **2022**, *20*, 2681-2692.
36. Mazur-Marzec, H.; Kaczkowska, M.J.; Blaszczyk, A.; Akcaalan, R.; Spoof, L.; Merilluto, J. *Mar. Drugs* **2013**, *11*, 1-19.
37. Sanz, M.; Andreote, A.P.D.; Fiore, M.F.; Dorr, F.A.; Pinto, E. *Mar. Drugs* **2015**, *13*, 3892-3919.

Figure S1. ^{1}H NMR Spectrum of Aeruginosin KT688 (**1**) in DMSO-d_6

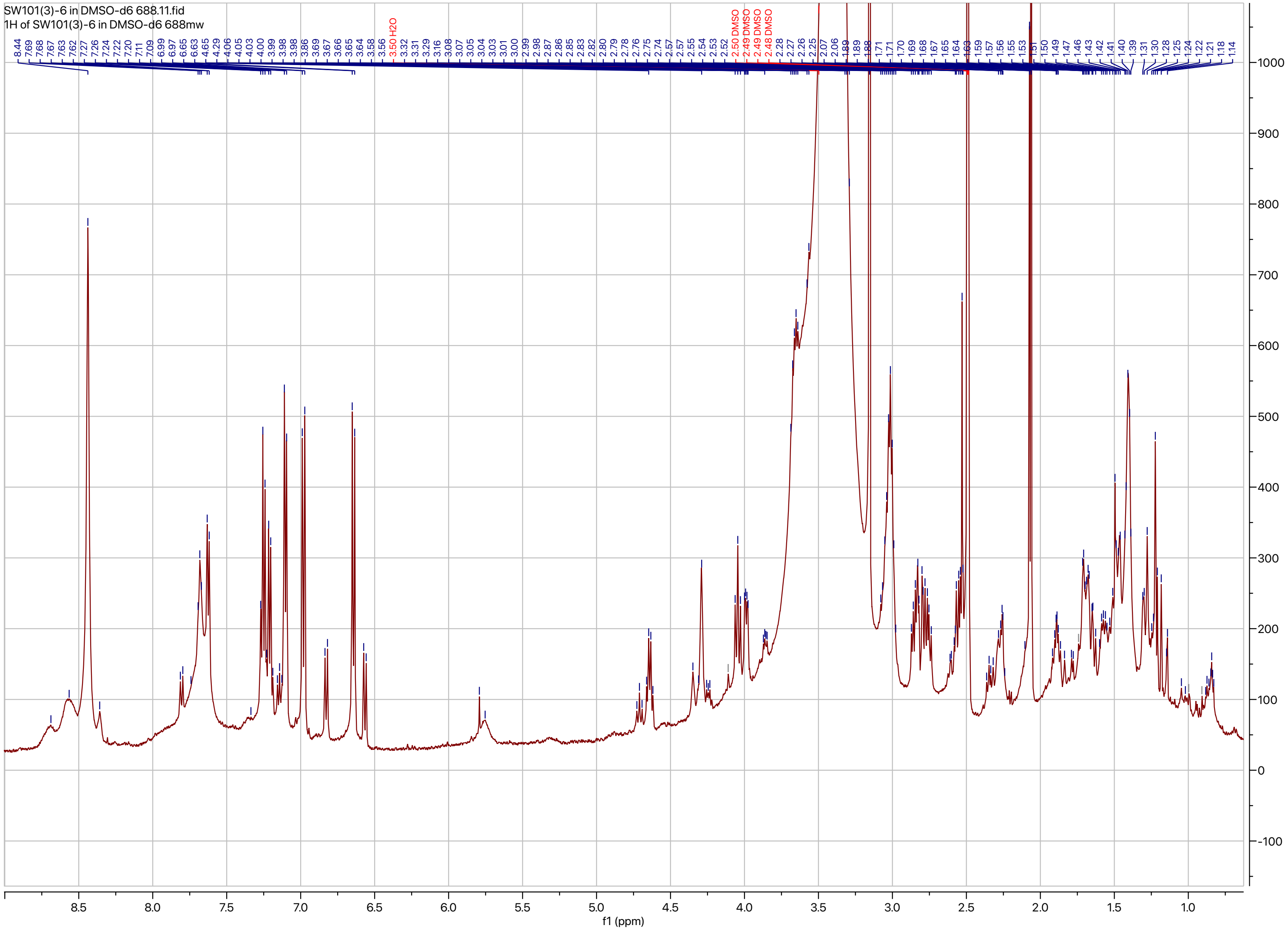

Figure S2. ^{13}C NMR Spectrum of Aeruginosin KT688 (**1**) in DMSO-d_6

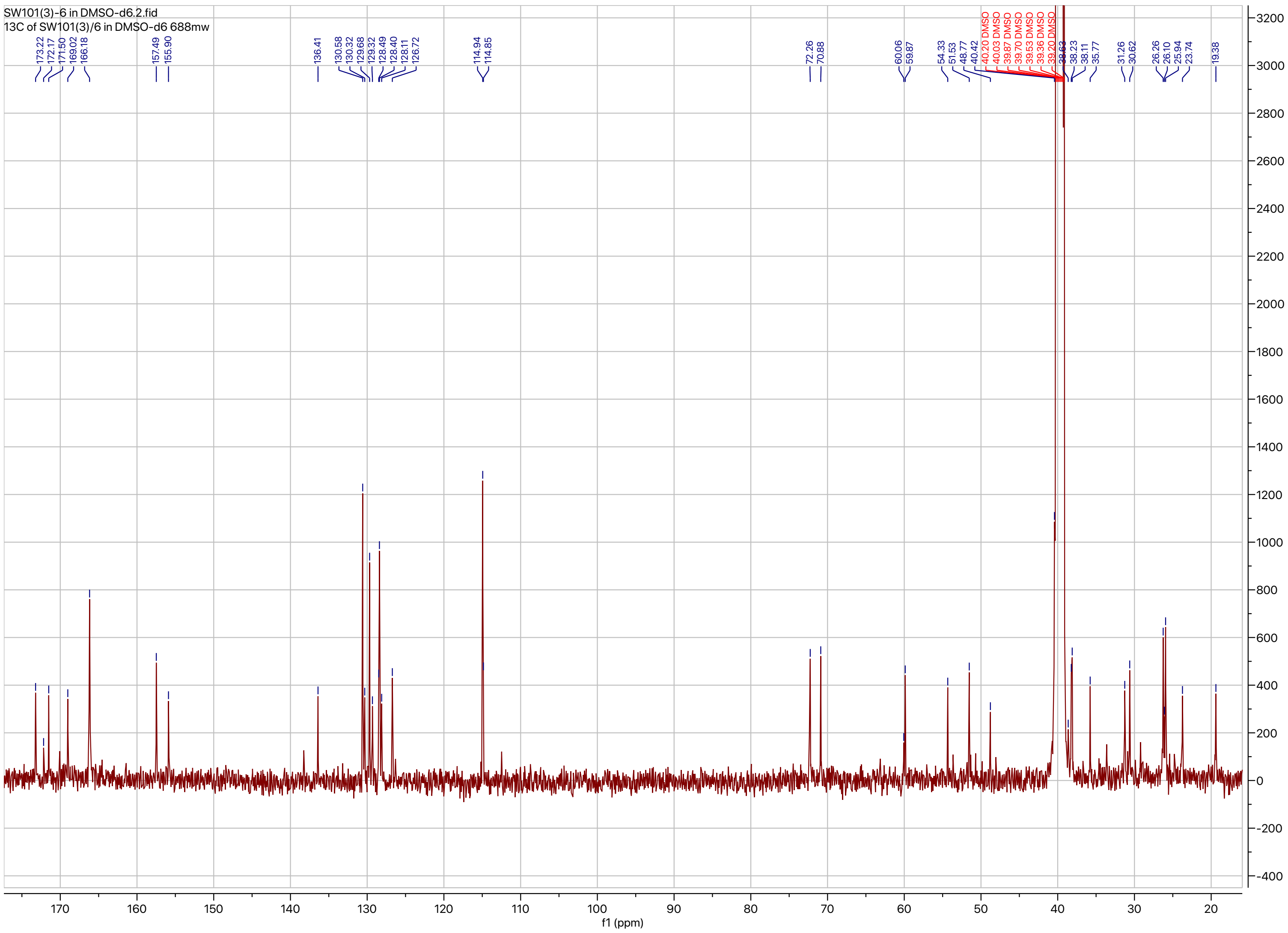

Figure S3. HSQC Spectrum of Aeruginosin KT688 (**1**) in DMSO-d_6

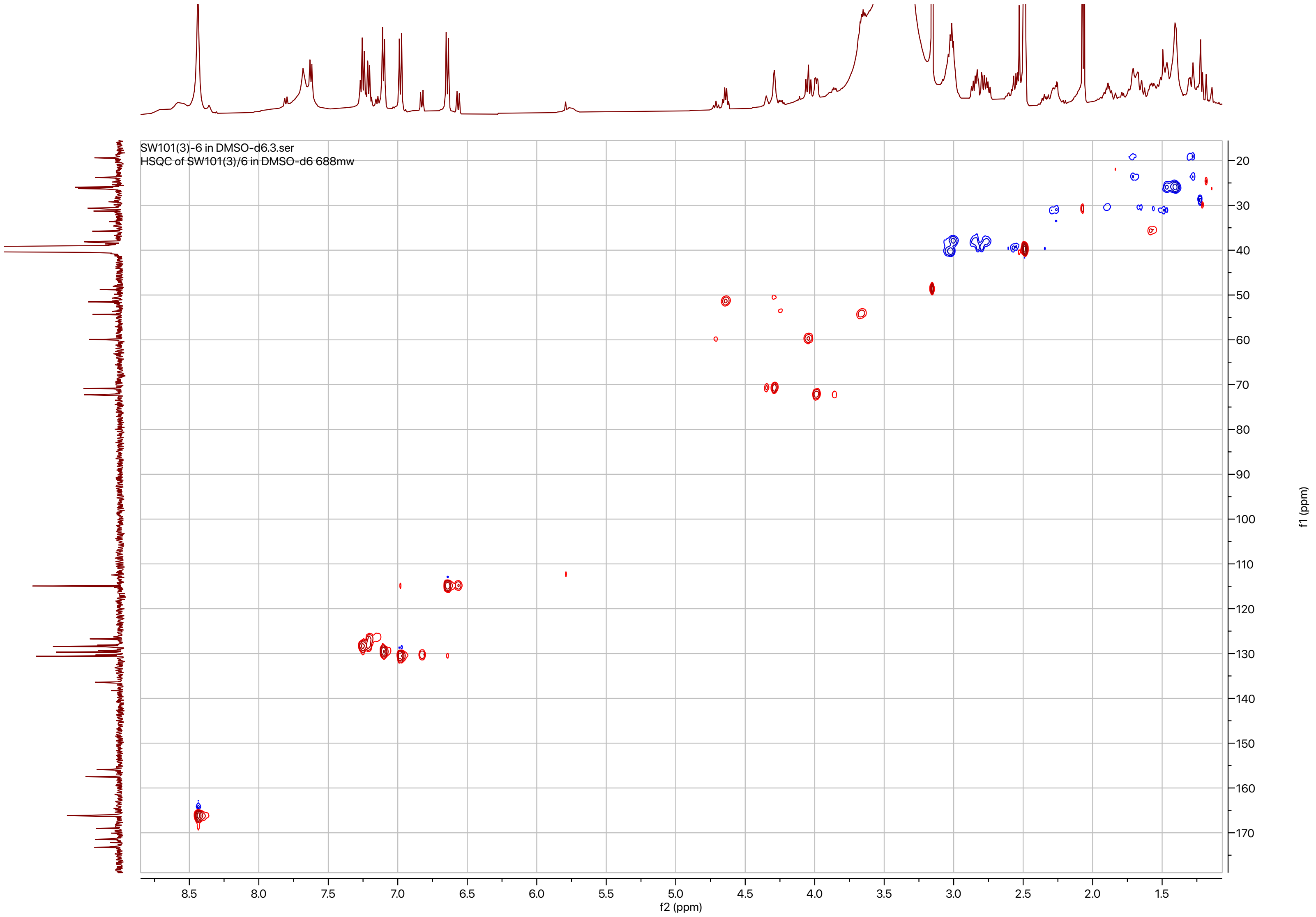

Figure S4. HMBC Spectrum of Aeruginosin KT688 (**1**) in DMSO-d_6

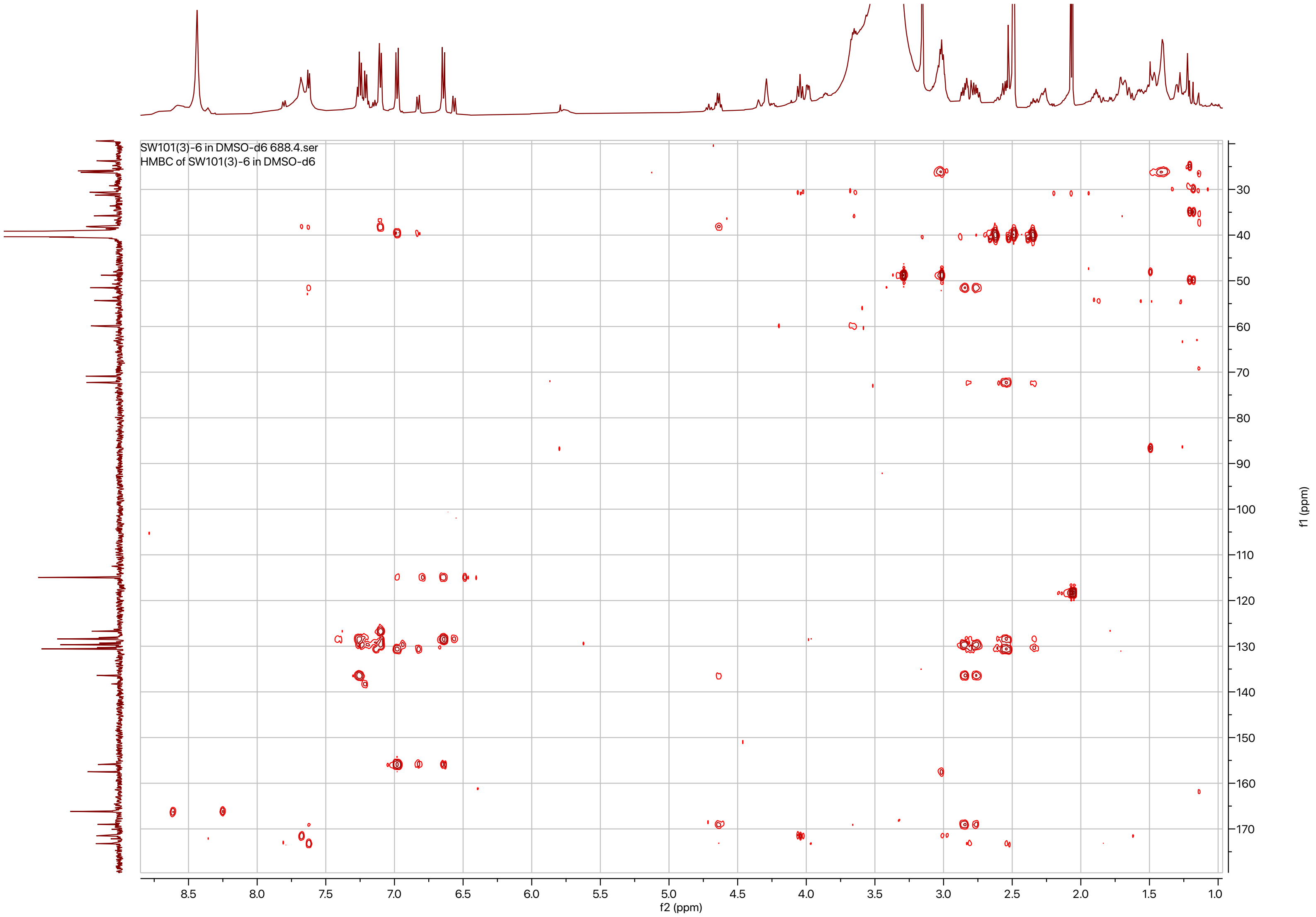

Figure S5. COSY Spectrum of Aeruginosin KT688 (**1**) in DMSO-d_6

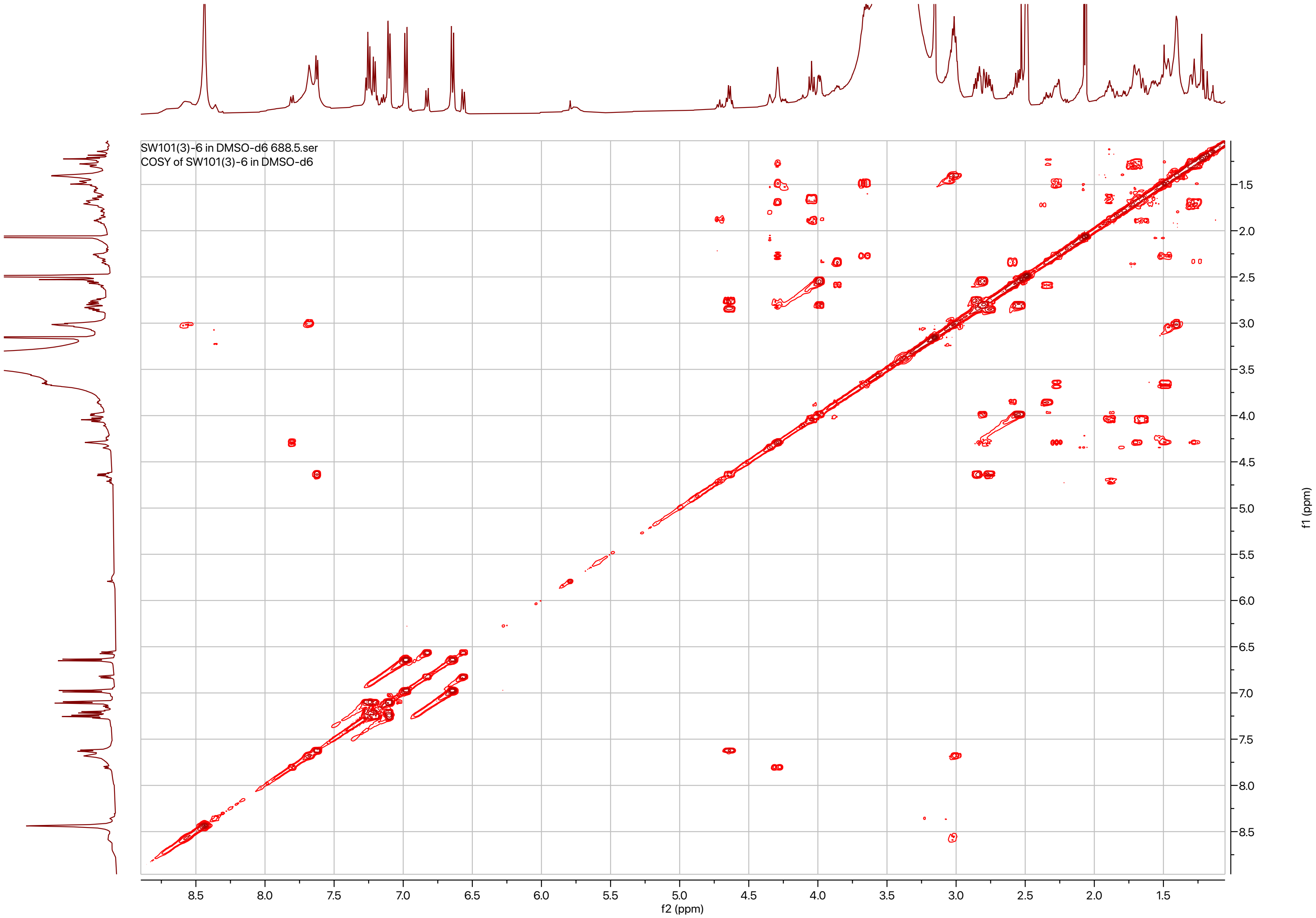

Figure S6. TOCSY Spectrum of Aeruginosin KT688 (**1**) in DMSO-d_6

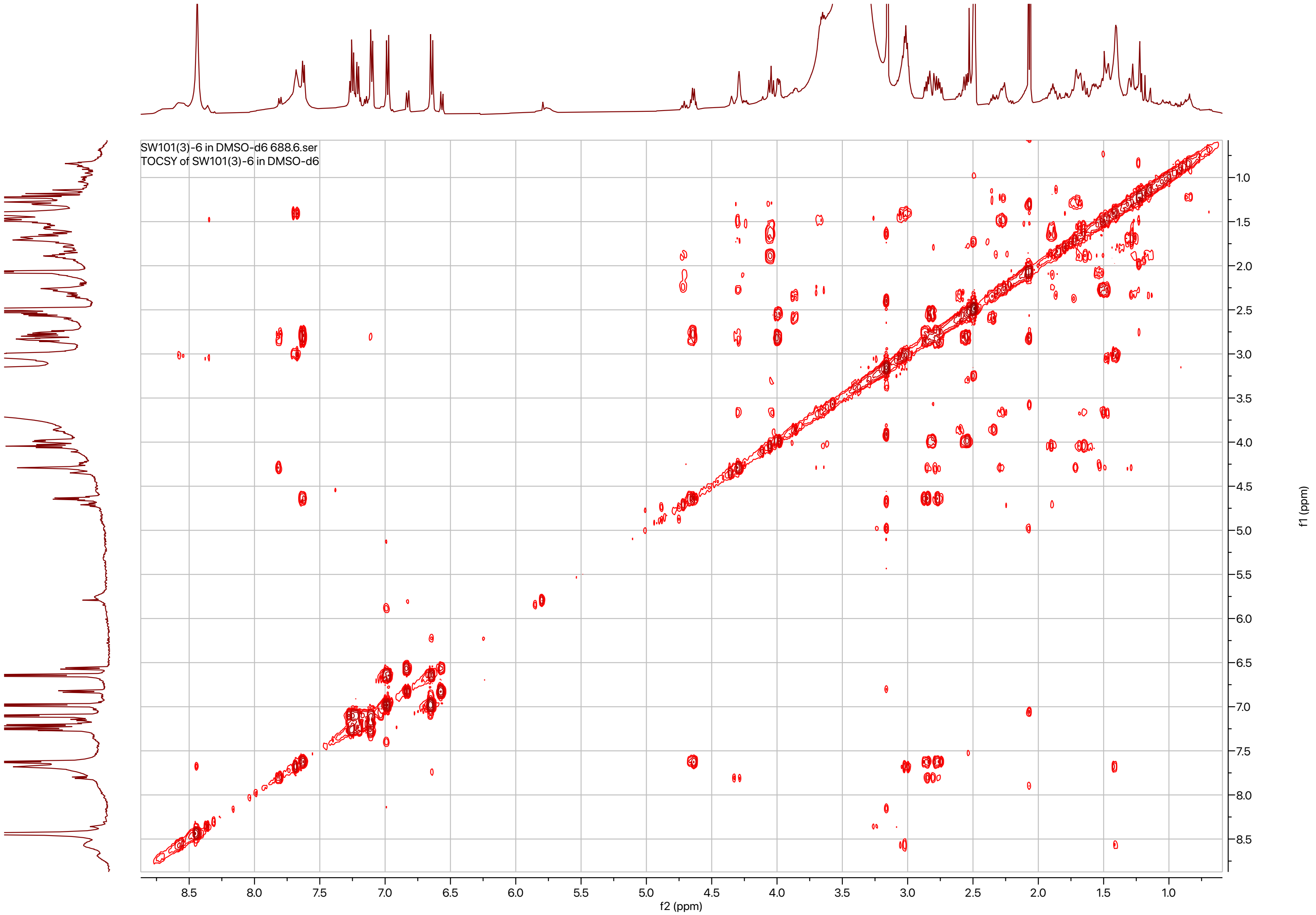

Figure S7. ROESY Spectrum of Aeruginosin KT688 (**1**) in DMSO-d_6

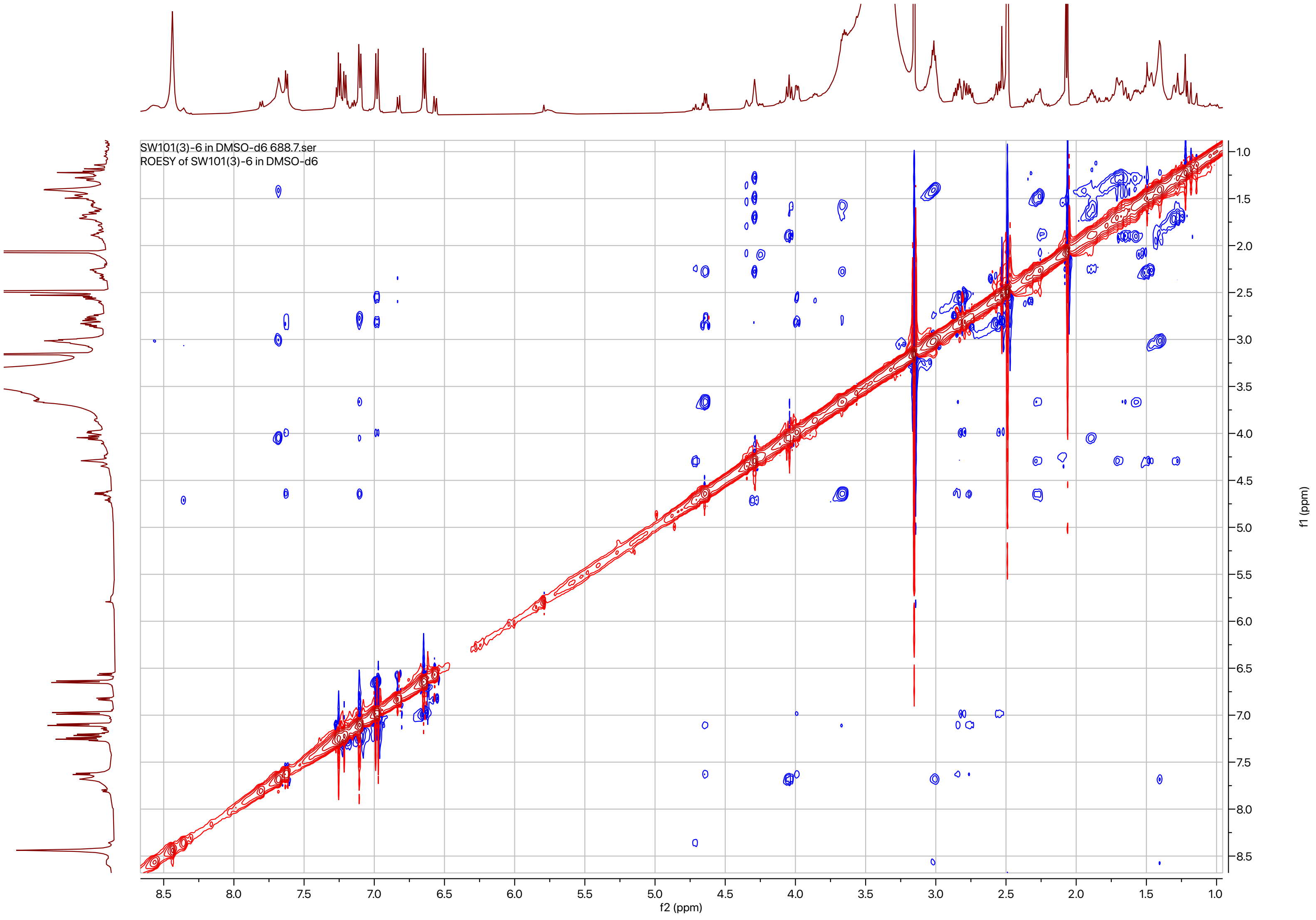

Table S2. NMR Data of the Minor *cis* Rotamer of Aeruginosin KT688 (**1**) in DMSO-d_6[a]

Position	δ_C mult.	δ_H mult. (J in Hz)	HMBC Correlation	COSY Correlation	TOCSY Correlation	ROESY Correlation
Hpla 1	173.1, C		Phe-2-NH, Hpla-3a			
2	72.4, CH	3.86, dd (7.8, 4.2)	Hpla-3a,3b	Hpla-3a,3b	Hpla-3a,3b	Phe-2-NH, Hpla-3a,3b,5,5’
2-OH		5.75, brm				
3a	39.7, CH_2	2.59, m	Hpla-5,5’	Hpla-2,3b	Hpla-2,3b	Hpla-2,5,5’ Hpla-5,5’
3b		2.34, m		Hpla-2,3a	Hpla-2,3a	
4	128.4, C		Hpla-3a,3b,6,6’			
5,5’	130.3, CH x2	6.83, d (8.2)	Hpla-3a,3b,6,6’	Hpla-6,6’	Hpla-6,6’	Hpla-2,3a,3b
6,6’	114.8, CH x2	6.56, d (8.2)	Hpla-6’,6	Hpla-5,5’	Hpla-5,5’	Phe-5,5’
7	155.8, C		Hpla-5,5’,6,6’			
Phe 1	170.1, C		Phe-2-NH			
2	50.7, CH	4.29, m		Phe-2-NH, 3a,3b	Phe-2-NH, 3a,3b	Phe-2-NH, 3a,3b,5,5’, ChoiSul-2
2-NH		7.81, d (8.5)		Phe-2,	Phe-2,3a,3b,	Phe-2, Hpla-2
3a	36.7, CH_2	2.83, m	Phe-2		Phe-2-NH,	Phe-2
3b		2.78, m	Phe-2		Phe-2-NH,	Phe-2
4	138.3, C		Phe-2-NH,3a,3b			
5,5’	129.3, CH x2	7.10, d (7.3)	Phe-3a,3b,5’,5	Phe-6,6’	Phe-6,6’	Phe-2
6,6’	128.1, CH x2	7.23, m	Phe-6,6,	Phe-5,5’	Phe-5,5’	ChoiSul-2
7	126.3, CH	7.14, t (7.3)	Phe-5,5’	Phe-6,6’	Phe-6,6’	
ChoiSul 1	172.2, C		Agm-1-NH			
2	60.1, CH	4.71, t (8.9)		ChoiSul-3pe,3pa	ChoiSul-3pe,3pa,7a	Phe-2,6,6’, ChoiSul-3pe,7a

3pe[b]	33.6, CH_2	2.25, m		ChoiSul-2,3pa		ChoiSul-2,3pe,3pa, Agm-2<br>ChoiSul-3a
3pa[b]		1.88, m		ChoiSul-2,3pe		
3a	34.5, CH	2.12, m				
4a	19.5, CH_2	1.95, m		ChoiSul-5a,5b,		
4b		1.41, m		ChoiSul-4a,5a		
5a	23.9, CH_2	1.80, m		ChoiSul-5b,6	ChoiSul-5b	ChoiSul-5a,6,
5b		1.37, m		ChoiSul-5a,6	ChoiSul-5a	
6	70.9, CH	4.34, brs		ChoiSul-5a,5b,<br>7pa,7pe	ChoiSul-7pa	ChoiSul-5a,5b,7pe,7pa
7pe	34.6, CH_2	2.10, m		ChoiSul-5a,7a	ChoiSul-5a,7pa	ChoiSul-7pa,7a
7pa		1.53, m			ChoiSul-7pa	
7a	53.7, CH	4.25, dt (11.0,<br>6.5)			ChoiSul-7pa,7pe	ChoiSul-7pe
Agm-1-<br>NH		8.36, t (5.2)		Agm-1a,1b	Agm-1a,1b,2	Agm-1a,1b,2
1a	38.4, CH_2	3.24, m		Agm-1b,2	Agm-1-NH,1b,2<br>Agm-1-NH,1a,2	Agm-1-NH,1b,2
1b		3.06, m		Agm-1a,2		Agm-1-NH,1a,2
2	26.1, CH_2	1.47, m	Agm-1a,3		Agm-1-NH	Agm-1-NH
3	26.4, CH_2	1.41, m	Agm-2			
4	40.4 CH_2	3.02		Agm-4-NH	Agm-4-NH	
4-NH		8.72, brs		Agm-4	Agm-4	
5	157.6, C					
5-NH,NH_2		8.69, brs<br>8.72, brs				

[a]500 MHz for ^{1}H, 125 MHz for ^{13}C. [b]pe: pseudo-equatorial, pa: pseudo-axial.

Figure S8. Positive HR ESI MS/MS Spectrum of Aeruginosin KT688 (**1**)

Single Mass Analysis
Tolerance = 10.0 PPM / DBE: min = -1.5, max = 50.0
Element prediction: Off
Number of isotope peaks used for i-FIT = 2
Monoisotopic Mass, Even Electron Ions
368 formula(e) evaluated with 5 results within limits (all results (up to 1000) for each mass)
Elements Used:

Mass	Calc. Mass	mDa	PPM	DBE	Formula	i-FIT	i-FIT Norm	Fit Conf %	C	H	N	O	Na	S
711.2802	711.2788	1.4	2.0	13.5	C32 H44 N6 O9 Na S	452.2	0.125	88.25	32	44	6	9	1	1
	711.2754	4.8	6.7	18.5	C35 H40 N6 O9 Na	455.2	3.117	4.43	35	40	6	9	1	
	711.2750	5.2	7.3	12.5	C35 H48 N2 O8 Na S2	455.6	3.470	3.11	35	48	2	8	1	2
	711.2862	-6.0	-8.4	12.5	C34 H48 N4 O7 Na S2	455.9	3.827	2.18	34	48	4	7	1	2
	711.2867	-6.5	-9.1	18.5	C34 H40 N8 O8 Na	456.0	3.896	2.03	34	40	8	8	1	

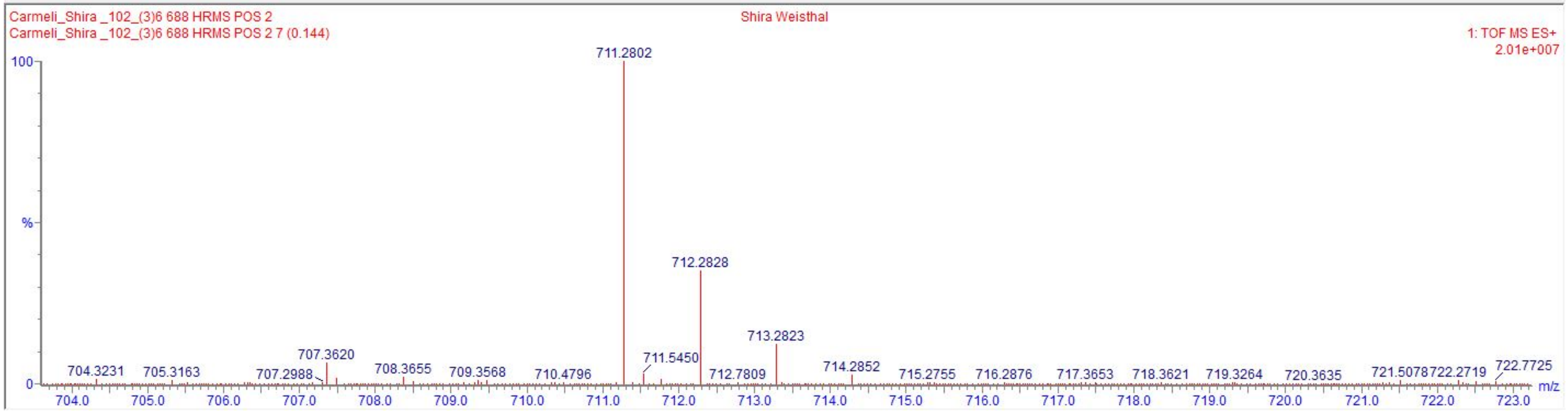

Table S3. Comparison of the chemical shifts of the Choi-6-sulfate moiety of the *trans* rotamer of Aeruginosins KT688 (**1**) and GE766.

	Aeruginosins KT688 (**1**)		Aeruginosins GE766	
Position	δ_C, mult.	δ_H, mult. (*J* in Hz)	δ_C, mult.	δ_H, mult. (*J* in Hz)
Choi-1	171.5, C		171.5, C	
Choi-2	59.9, CH	4.05, t (9.1)	59.9, CH	4.16, t (9.2)
Choi-3pe[a]	30.6, CH_2	1.89, m*	30.6, CH_2	2.02, m
Choi-3pa[a]		1.66, m		1.74, m
Choi-3a	35.8, CH	1.58, m*	35.9, CH	2.28, m
Choi-4a	19.4, CH_2	1.71, m	19.3, CH_2	1.98, m
4b		1.29, m		1.42, m
Choi-5a	23.7, CH_2	1.70, m	23.3, CH_2	1.85, m
5b		1.28, m		1.32, m
Choi-6-sulfate	70.9, CH	4.29, brs	70.8, CH	4.35, brs
Choi-7pe	31.3, CH_2	2.27, m	31.6, CH_2	2.24, m
7pa		1.49, m		1.68, m
Choi-7a	54.3, CH	3.66, dt (11.1, 5.8)	54.0, CH	4.00, dt (10.0, 4.8)

*The anisotropic effect on these protons is most probably due to the proximity of the phenyl moiety of the adjacent Phenylalanyl residue. [a] pe: pseudo-equatorial, pa: pseudo-axial.

Table S4. NMR Data of the Major *cis* Rotamer of Aeruginosins KT608A, KT608B and Minor *cis* KT688 (**1**) in DMSO-d_6[a]

	Aeruginosin KT608A		Aeruginosins KT608B		Aeruginosins KT688 (**1**)	
Position	δ_C, mult.[b]	δ_H, mult., *J* (Hz)	δ_C, mult.[b]	δ_H, mult., *J* (Hz)	δ_C, mult.[b]	δ_H, mult., *J* (Hz)
Agm 1	38.6, CH_2	3.18, m<br>3.06, m	38.6, CH_2	3.07, m<br>3.19, m	38.4, CH_2	3.24, m<br>3.06, m
2	26.2, CH_2	1.45, m	26.2, CH_2	1.45, m	26.1, CH_2	1.47, m
3	26.4, CH_2	1.41, m	26.4, CH_2	1.41, m	26.4, CH_2	1.41, m
4	40.6, CH_2	3.08, m	40.5, CH_2	3.07, m	40.4, CH_2	3.02, m
5	156.9, C		156.9, C		157.6, C	
1-NH		8.29, t (5.5)		8.20, t (5.2)		8.36, t (5.2)
4-NH		7.52, t (5.5)		7.62, brs		8.72, brs
Choi 1	172.3, C		172.3, C		172.2, C	
2	59.6, CH	4.82, d (9.5)	59.5, CH	4.80, d (8.8)	60.1, CH	4.71, t (8.9)
3	33.6, CH_2	2.38, m (ax)<br>1.70, dd (12.0,6.0)	33.8, CH_2	2.35, m (ax)<br>1.70, dd (12.0,6.0)	33.6, CH_2	2.25, m<br>1.88, m
3a	**32.7, CH**	**2.23, m**	**32.7, CH**	**2.22, m**	**34.5, CH**	**2.12, m**
4	**22.7, CH_2**	**1.63, m (2H)**	**22.7, CH_2**	**1.60, m (2H)**	**19.5, CH_2**	**1.95, m**<br>**1.41, m**
5	**29.9,** CH_2	**1.56, m (eq)**<br>**1.19, m (ax)**	**29.9,** CH_2	**1.55, m (eq)**<br>**1.17, m (ax)**	**23.9, CH_2**	**1.80, m**<br>**1.37, m**
6	**67.0, CH**	**3.30, m**	**67.0, CH**	**3.30, m**	**70.9, CH**	**4.34, m**
7	**36.1, CH_2**	**2.37, m (eq)**<br>**0.83, q (12.0)**	**36.2, CH_2**	**2.35, m (eq)**<br>**0.83, q (12.0)**	**34.6, CH_2**	**2.10, m**<br>**1.53, m**

7a	56.8, CH	4.04, ddd (12.0,8.0,6.5)	56.8, CH	4.05, ddd (12.0,8.2,5.6)	53.7, CH	4.25, dt (11.0,6.5)
Phe 1	170.0, C		170.0, C		170.1, C	
2	51.8, CH	4.20, m	51.4, CH	4.26, m	50.7	4.29, m
3	36.5, CH_2	2.84, m<br>2.81, m	36.5, CH_2	2.80, m<br>2.81, m	36.7, CH_2	2.83, m<br>2.78, m
4	138.4, C		138.2, C		138.3, C	
5,5’	129.2, CH	7.12, d (7.0)	129.2, CH	7.10, d (7.0)	129.3, CH	7.10, d (7.3)
6,6’	128.2, CH	7.19, t (7.0)	128.2, CH	7.22, m	128.1, CH	7.23, m
7	126.4, CH	7.14, t (7.0)	126.4, CH	7.16, m	126.3, CH	7.14, t (7.3)
NH		7.97, d (8.0)		7.97, d (8.0)		7.81, d (8.5)
Hpla 1	173.5, C		173.4, C		173.1, C	
2	72.0, CH	3.85, dd (9.0,3.5)	72.3, CH	3.88, m	72.4, CH	3.86, dd (7.8, 4.2)
3	39.5, CH_2	2.33, m<br>2.55, dd (14.0,3.5)	39.7, CH_2	2.29, m<br>2.60, dd (14.0,3.6)	39.7, CH_2	2.34, m<br>2.59, m
4	128.5, C		128.4, C		128.4, C	
5,5’	130.4, CH	6.82, d (8.4)	130.3, CH	6.85, d (8.4)	130.3, CH x2	6.83, d x2 (8.2)
6,6’	114.9, CH	6.57, d (8.4)	114.9, CH	6.58, d (8.4)	114.8, CH x2	6.56, d x2 (8.2)
7	155.7, C		155.7, C		155.8, C	
2-OH		5.05, brs		5.31, d 6.4		5.75, brm
7-OH		9.06, brs		9.10, brs		-

[a]400 MHz for ^1H, 100 MHz for ^{13}C for aeruginosins KT608A&B, 500 MHz for ^1H, 125 MHz for ^{13}C for aeruginosin KT688 (**1**).

Figure S9. LCMS Traces of Marfey's Method Amino Acids Analysis of Aeruginosin KT688 (**1**)

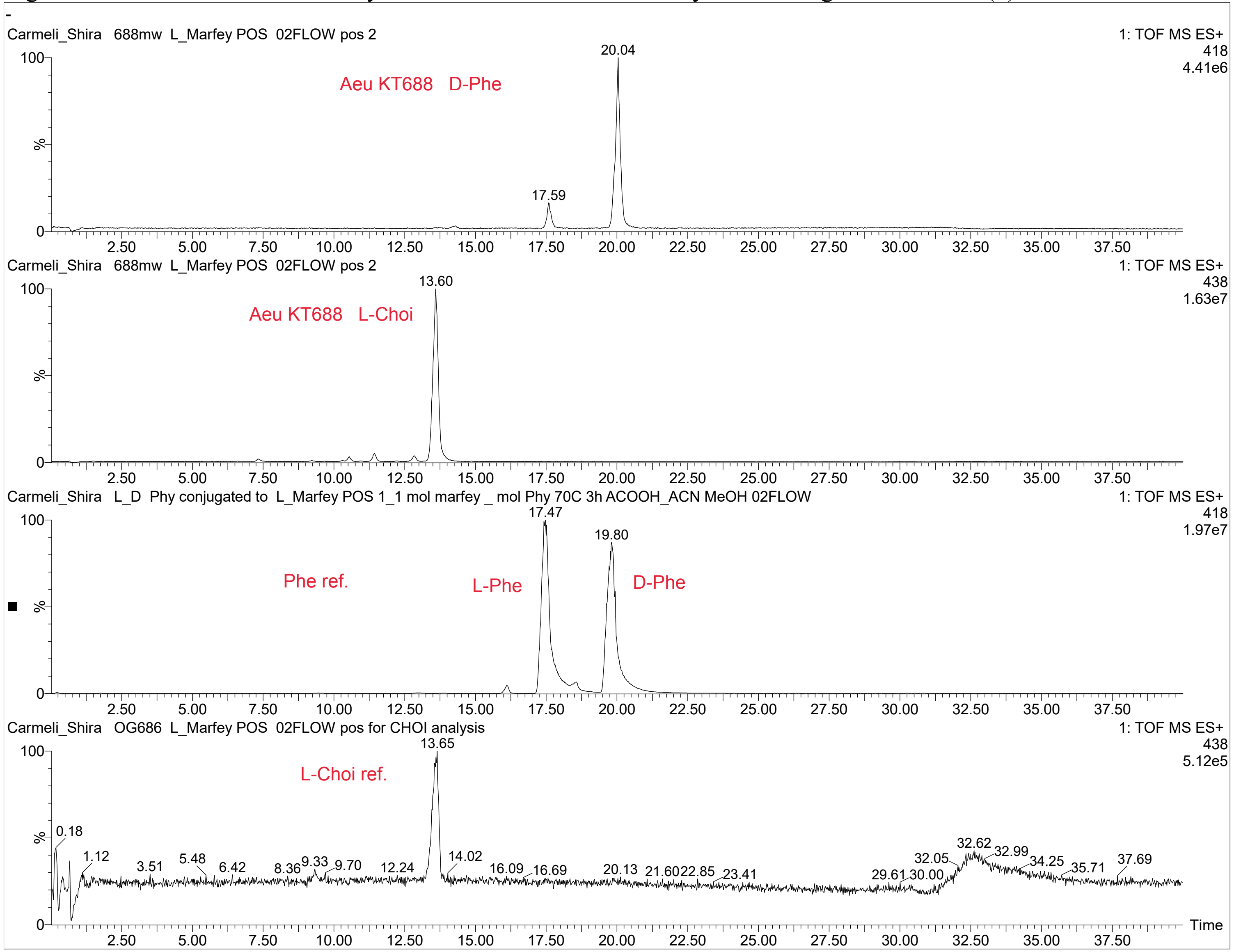

Figure S10. HPLC Traces of Chiral Chromatography of HPLA Analysis of Aeruginosin KT688 (**1**)

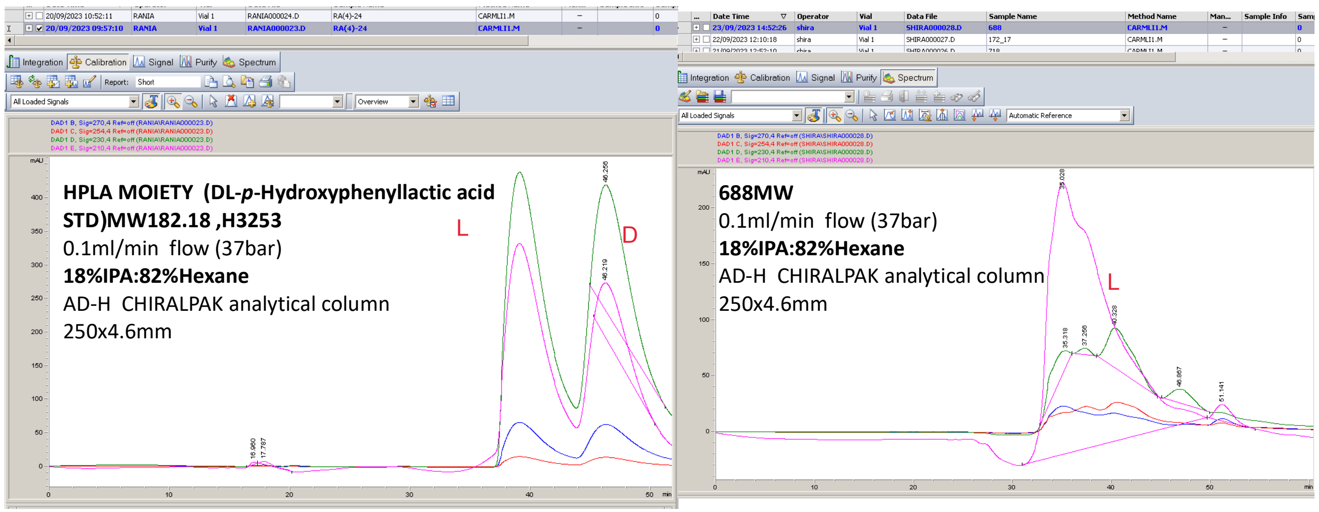

Figure S11. ^{1}H NMR Spectrum of Aeruginosin KT718 (**2**) in DMSO-d_6

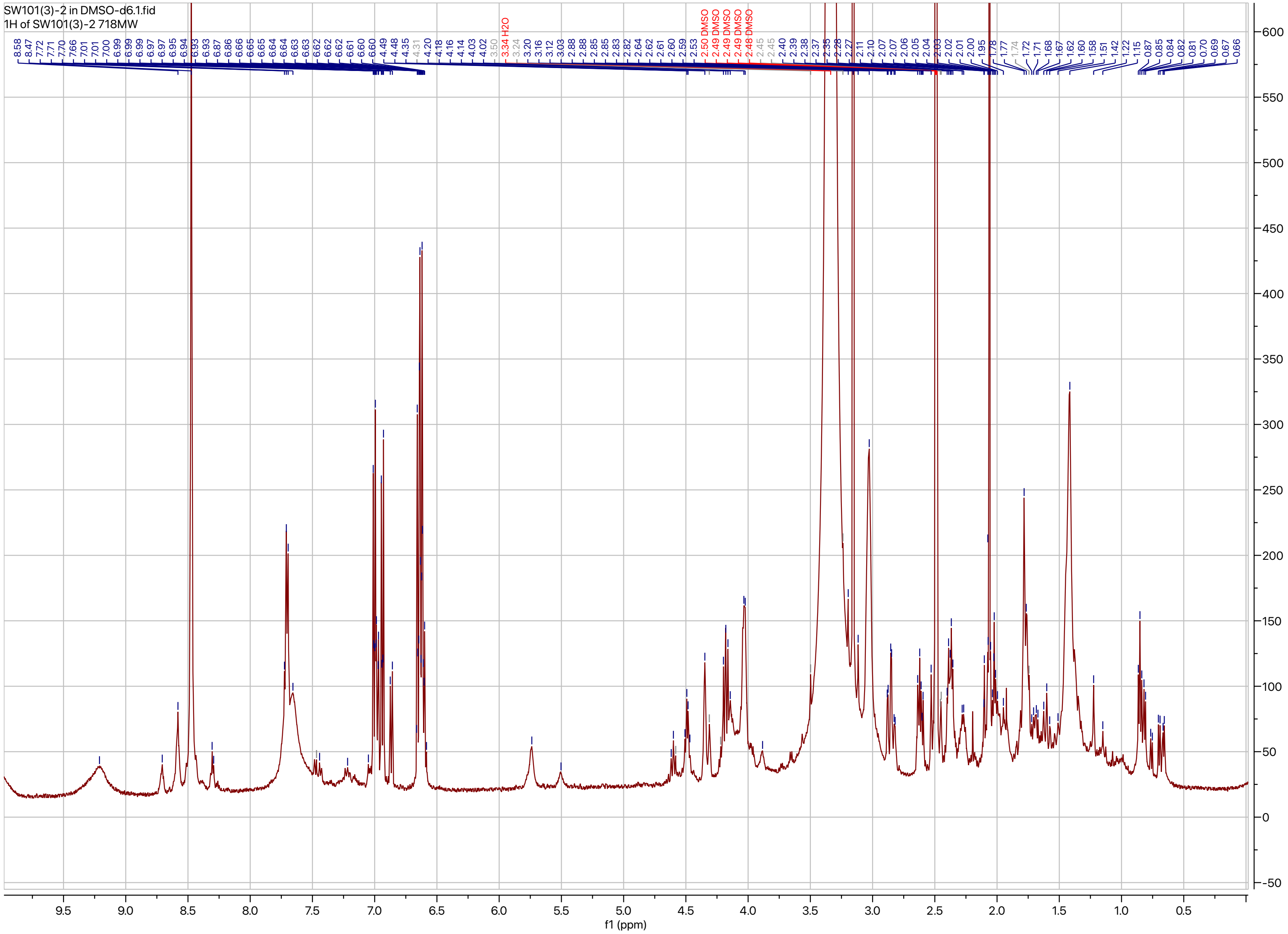

Figure S12. ^{13}C NMR Spectrum of Aeruginosin KT718 (**2**) in DMSO-d_6

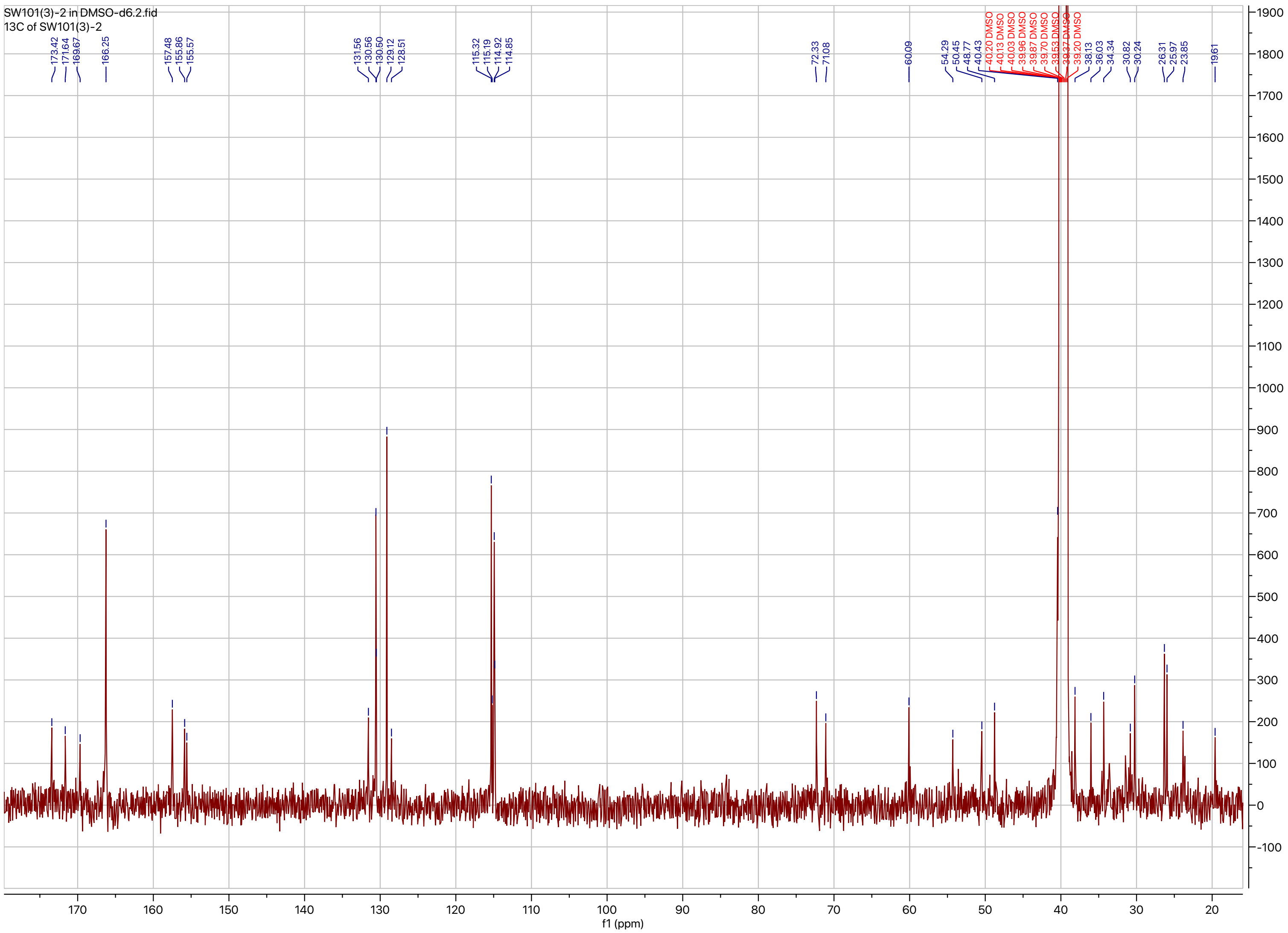

Figure S13. HSQC Spectrum of Aeruginosin KT718 (**2**) in DMSO-d_6

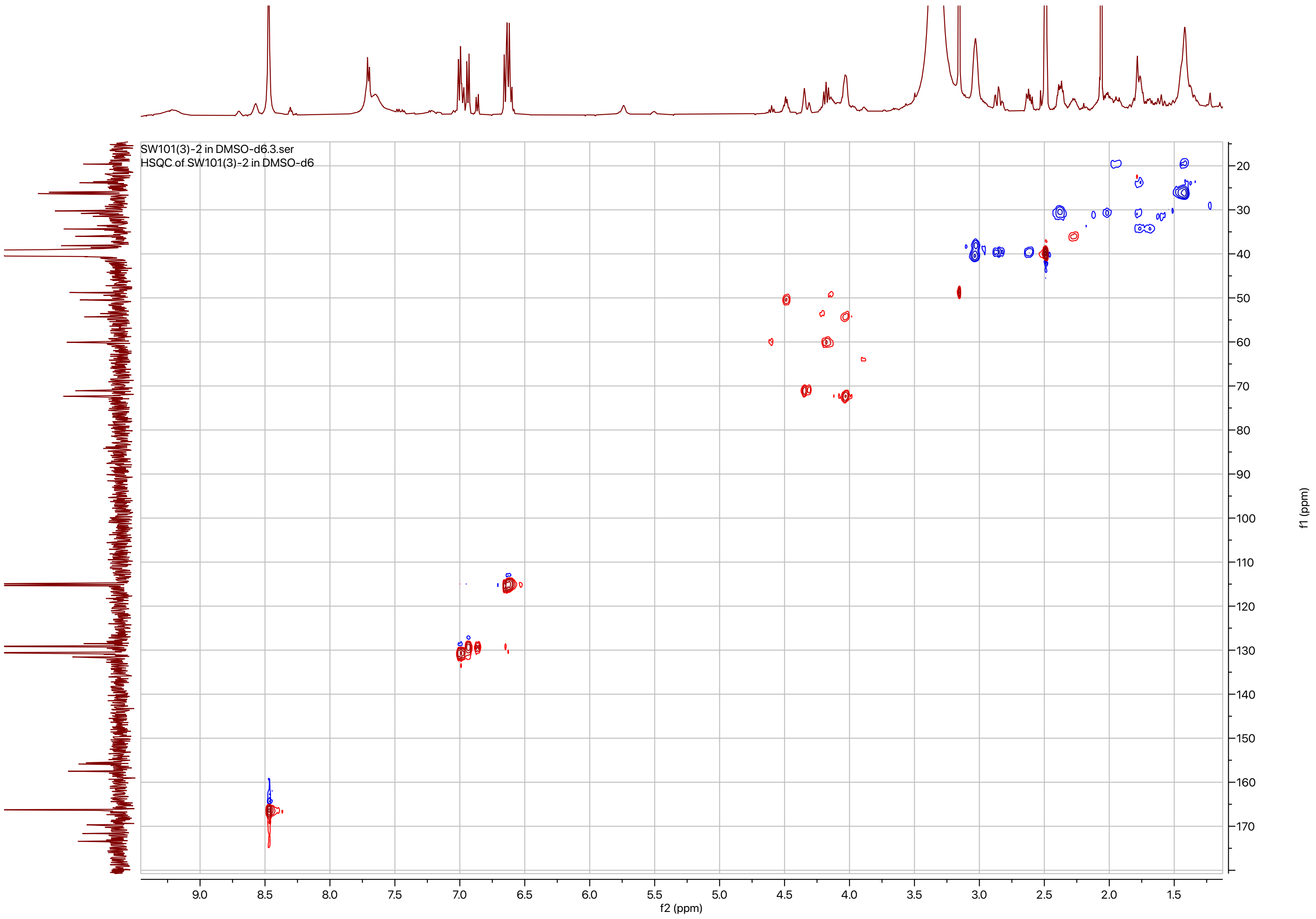

Figure S14. HMBC Spectrum of Aeruginosin KT718 (**2**) in DMSO-d_6

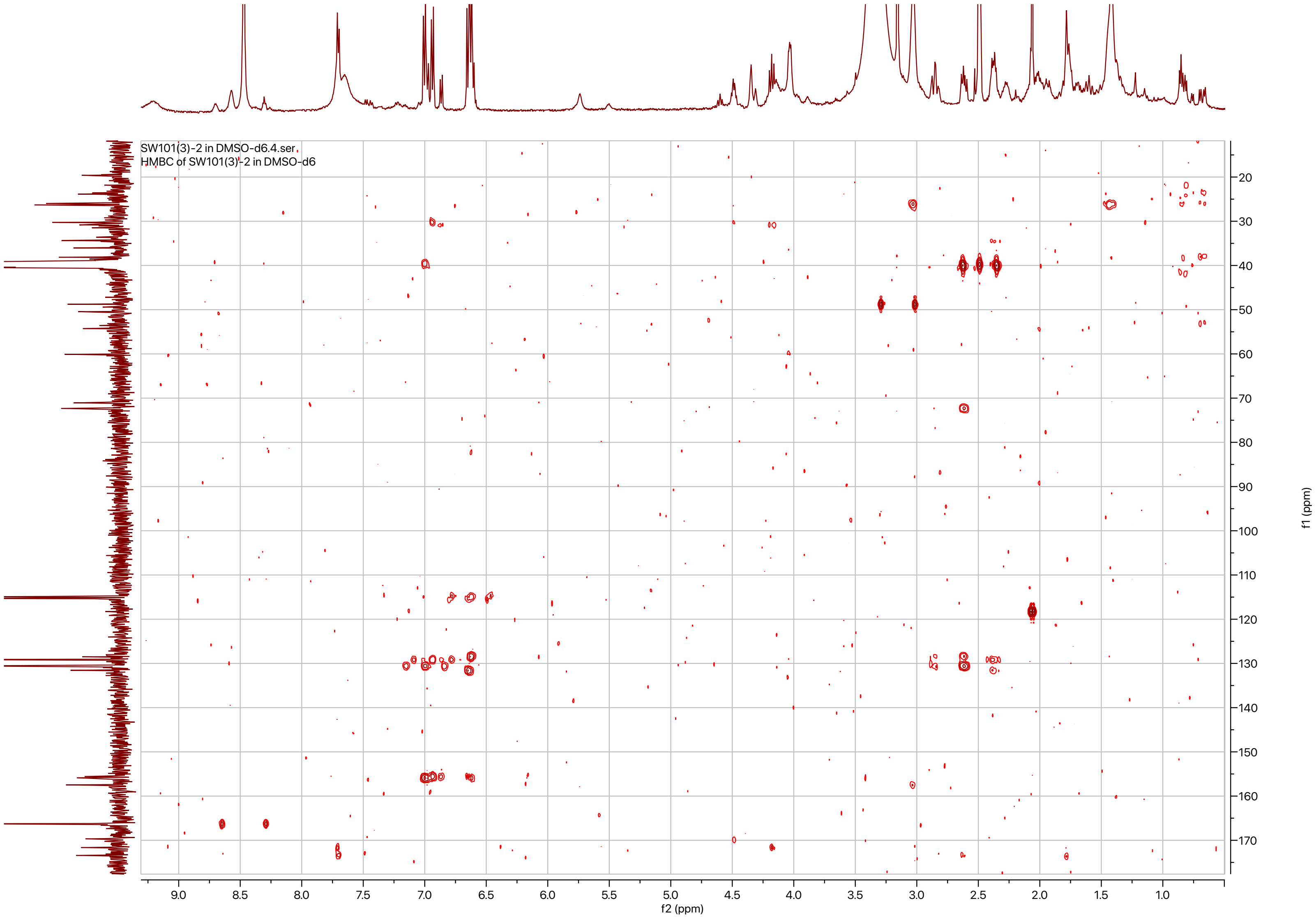

Figure S15. COSY Spectrum of Aeruginosin KT718 (**2**) in DMSO-d_6

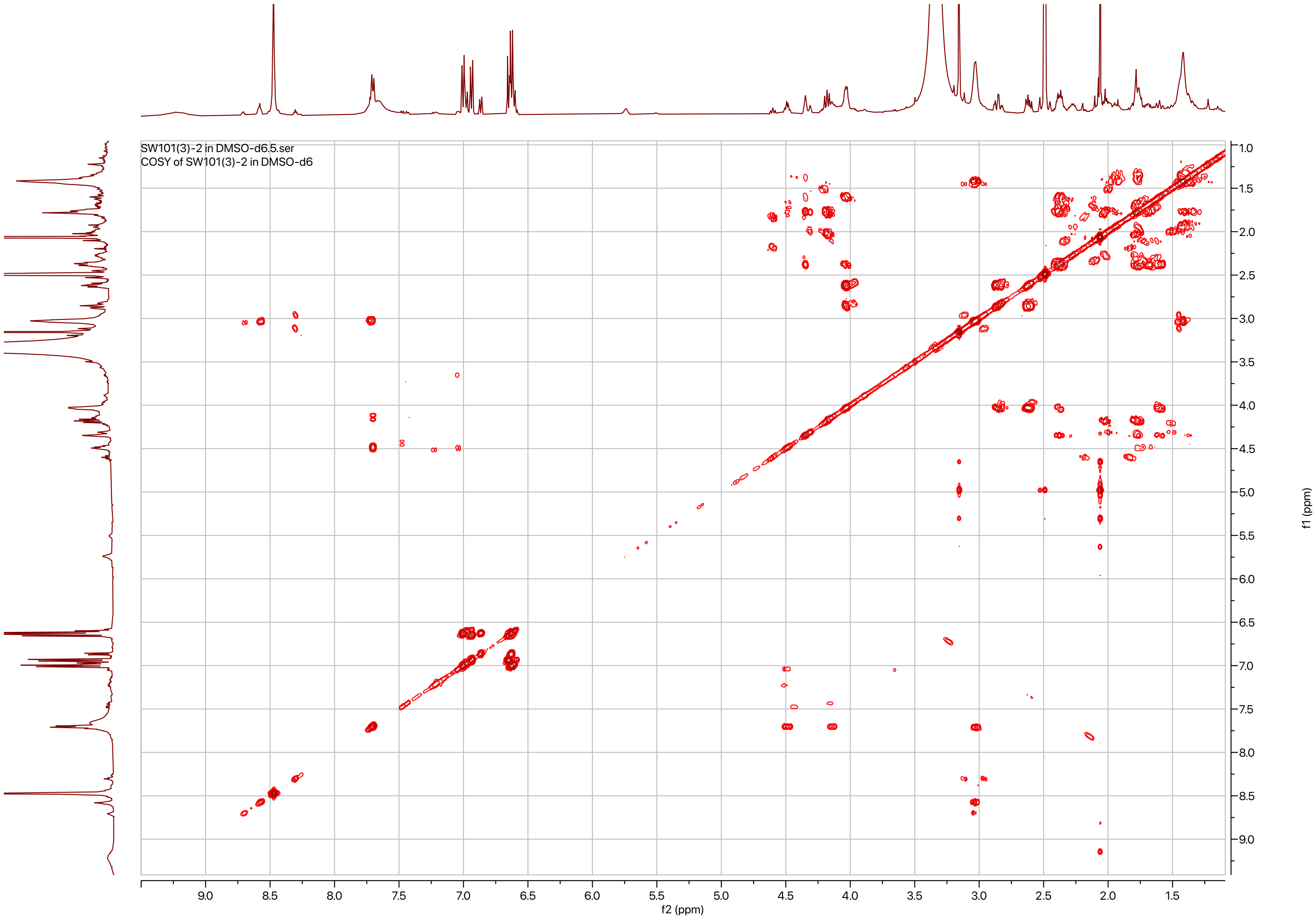

Figure S16. TOCSY Spectrum of Aeruginosin KT718 (**2**) in DMSO-d_6

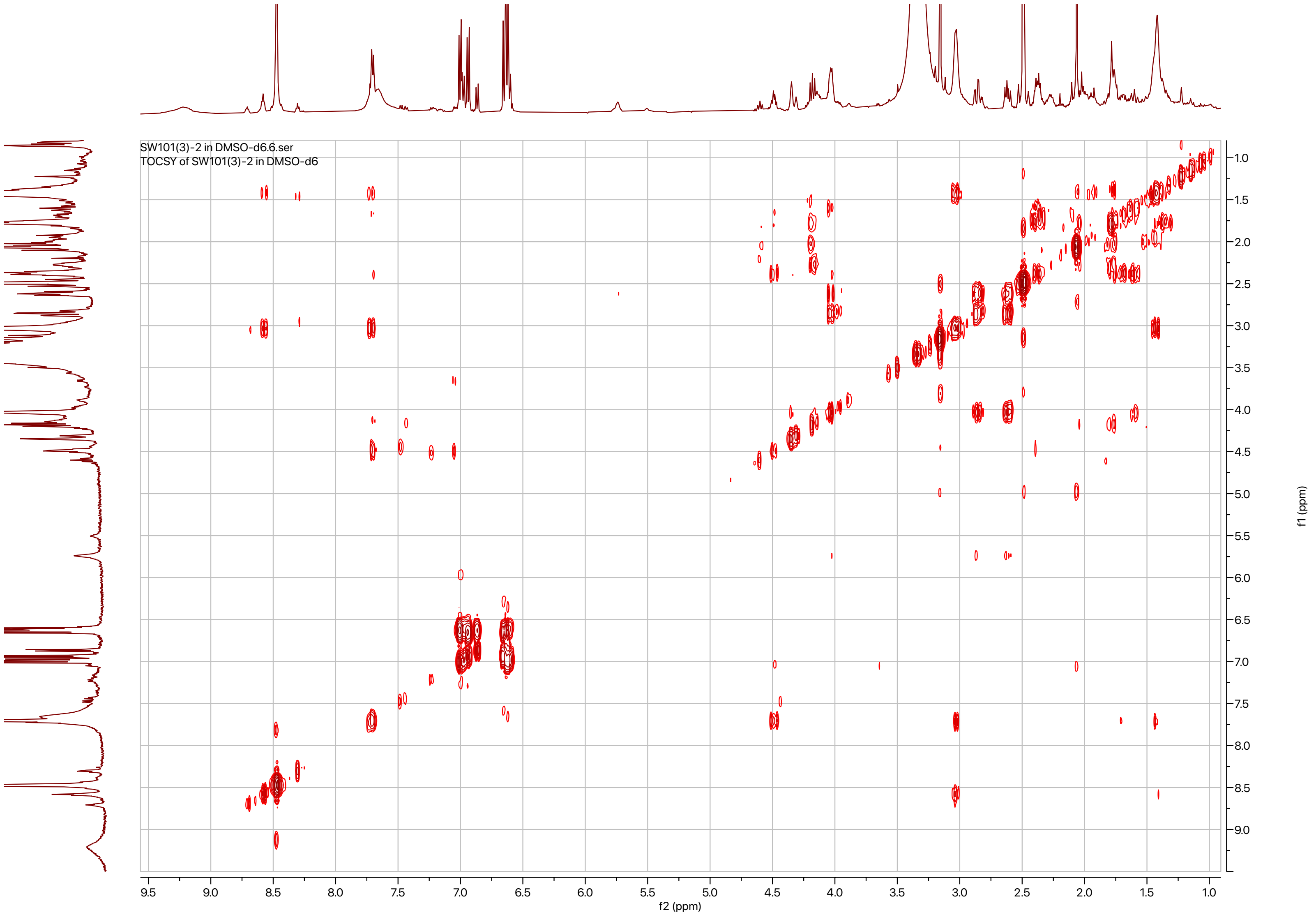

Figure S17. ROESY Spectrum of Aeruginosin KT718 (**2**) in DMSO-d_6

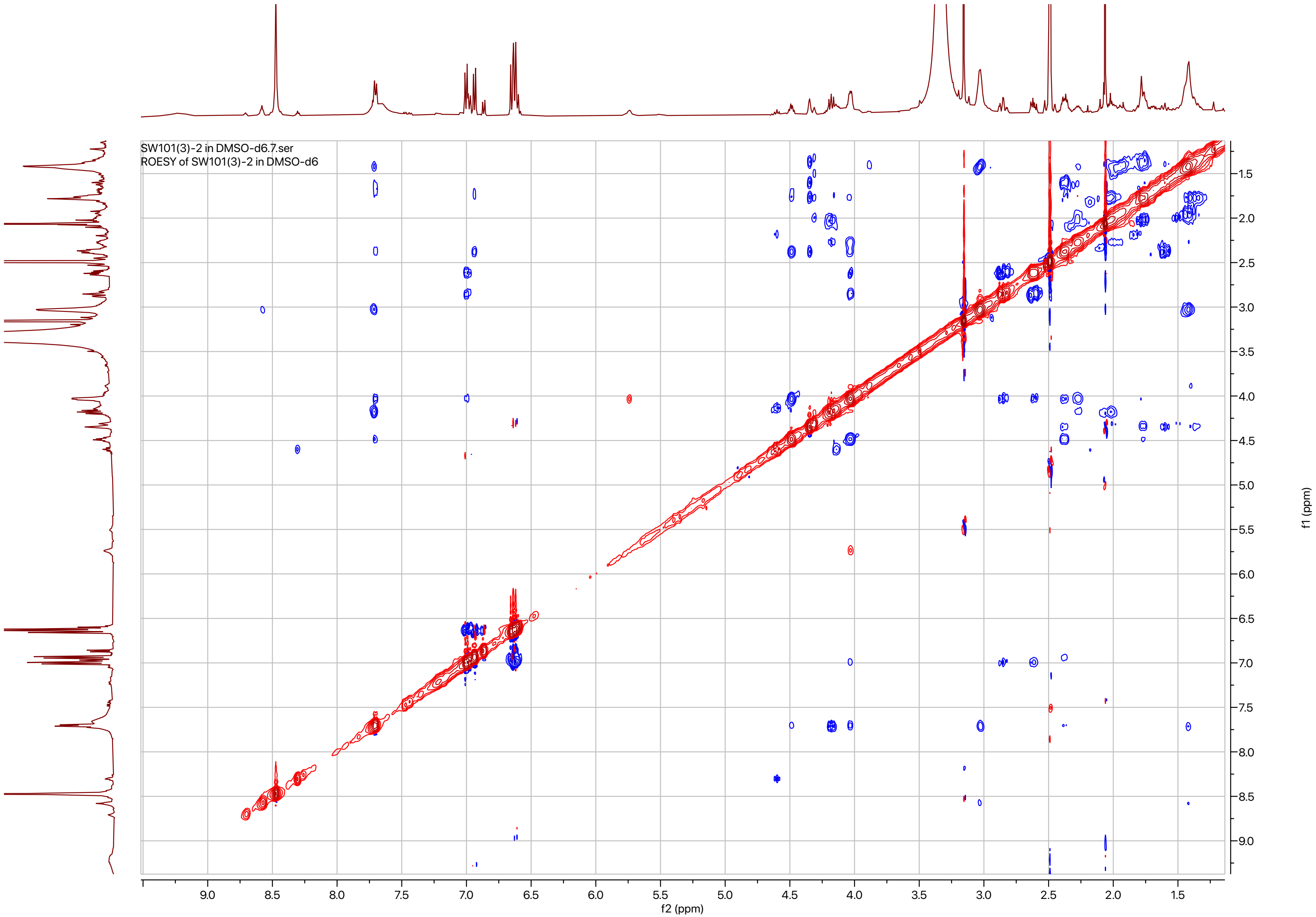

Table S5. NMR Data of Aeruginosin KT718 (**2**) minor *cis* rotamer in DMSO-d_6[a]

Position	δ_C, mult.	δ_H, mult. (*J* in Hz)	HMBC Correlation	COSY Correlation	TOCSY Correlation	ROESY Correlation
Hpla 1	173.0, C		Hty-2-NH, Hpla-3a,3b			
2	72.3, CH	3.98, m		Hpla-3a,3b		Hpla-3a,3b
2-OH		5.50, brs			Hpla-2,3a,3b	
3a	39.3, CH_2	2.84, dd (14.3,4.4)		Hpla-2,3b		
3b		2.61, dd (14.3,7.5)		Hpla-2,3a		
4	128.0, C		Hpla-3b			
5,5’	130.5, CH x2	6.98, d (8.6)	Hpla-3a,3b,5,5’	Hpla-6,6’		Hpla-2,3a,3b
6,6’	114.9, CH x2	6.61, d (8.6)	Hpla-6’,6	Hpla-5,5’		
7	155.9, C		Hpla-5,5’			
Hty 1	169.6, C					
2	49.4, CH	4.14, m		Hty-3eq,3ax		ChoiSul-2,3eq,3ax
2-NH		7.71, d (7.7)		Hty-2	Hty-2,3ax,4	
3eq	31.0, CH_2	2.11, m		Hty-2,3ax,4		
3ax		1.62, m		Hty-2,3eq,4		
4	30.5, CH_2	2.34, m		Hty-3eq,3ax		
5	132.1, C		Hty-4,7,7’			
6,6’	129.1, CH x2	6.86, d (8.6)	Hty-4,6’,6	Hty-7,7’		
7,7’	115.3, CH x2	6.63, d (8.6)	Hty-7’,7	Hty-6,6’		
8	155.7, C		Hty-6,6’			
ChoiSul 1	171.9, C		Hty-2-NH, ChoiSul-2,3ax			

2	60.0, CH	4.60, dd (9.1,8.6)		ChoiSul-3eq	ChoiSul-3eq	Hty-2, ChoiSul-3eq, Agm-1-NH
3eq	33.6, CH_2	2.18, m		ChoiSul-2,3ax,4		Hty-2
3ax		1.82, m		ChoiSul-2,3eq,4		
3a	34.6, CH	2.09, m		ChoiSul-3eq,3ax		
4eq	19.5, CH_2	1.93, m		ChoiSul-4ax,5eq,5ax		
4ax		1.42, m		ChoiSul-4eq,5eq,5ax		
5eq	23.6, CH_2	1.76, m		ChoiSul-4eq,4ax,5ax,6		
5ax		1.33, m		ChoiSul-4eq,4ax,5eq,6		
6	70.8, CH	4.31, brs		ChoiSul-5eq, 5ax,7eq,7ax		ChoiSul-5ax,7eq,7ax
7eq	30.2, CH_2	2.00, m		ChoiSul-6,7ax,7a		
7ax		1.51, m		ChoiSul-6,7eq,7a		
7a	53.6 CH	4.21, m		ChoiSul-7eq,7ax		
Agm-1-NH		8.31, t (5.5)		Agm-1a,1b	Agm-3	ChoiSul-2, Agm-1b,3
1a	38.5, CH_2	3.10, m		Agm-1-NH,1b,2		
1b		2.97, m		Agm-1-NH,1a,2		
2	26.1, CH_2	1.42, m	Agm-3,4	Agm-4		
3	26.0, CH_2	1.43, m	Agm-2,4			
4	40.4, CH_2	3.03, m		Agm-3		Agm-3
4-NH		8.71, dd (5.9,4.8)		Agm-4	Agm-4	
5	157.5, C		Agm-4			
5-NH_2,NH		7.65, brs				

[a]500 MHz for ^1H, 125 MHz for ^{13}C.

Figure S18. Positive HR ESI MS/MS Spectrum of Aeruginosin KT718 (**2**)

Single Mass Analysis
Tolerance = 10.0 PPM / DBE: min = -1.5, max = 50.0
Element prediction: Off
Number of isotope peaks used for i-FIT = 2
Monoisotopic Mass, Odd and Even Electron Ions
312 formula(e) evaluated with 6 results within limits (all results (up to 1000) for each mass)
Elements Used:

Mass	Calc. Mass	mDa	PPM	DBE	Formula	i-FIT	i-FIT Norm	Fit Conf %	C	H	N	O	Na	S
741.2906	741.2894	1.2	1.6	13.5	C33 H46 N6 O10 Na S	497.1	12.058	0.00	33	46	6	10	1	1
	741.2928	-2.2	-3.0	8.5	C30 H50 N6 O10 Na S2	485.0	0.000	100.00	30	50	6	10	1	2
	741.2954	-4.8	-6.5	13.0	C33 H48 N7 O7 Na S2	499.6	14.614	0.00	33	48	7	7	1	2
	741.2968	-6.2	-8.4	12.5	C35 H50 N4 O8 Na S2	501.1	16.140	0.00	35	50	4	8	1	2
	741.2842	6.4	8.6	13.0	C34 H48 N5 O8 Na S2	500.4	15.370	0.00	34	48	5	8	1	2
	741.2972	-6.6	-8.9	18.5	C35 H42 N8 O9 Na	501.2	16.155	0.00	35	42	8	9	1	

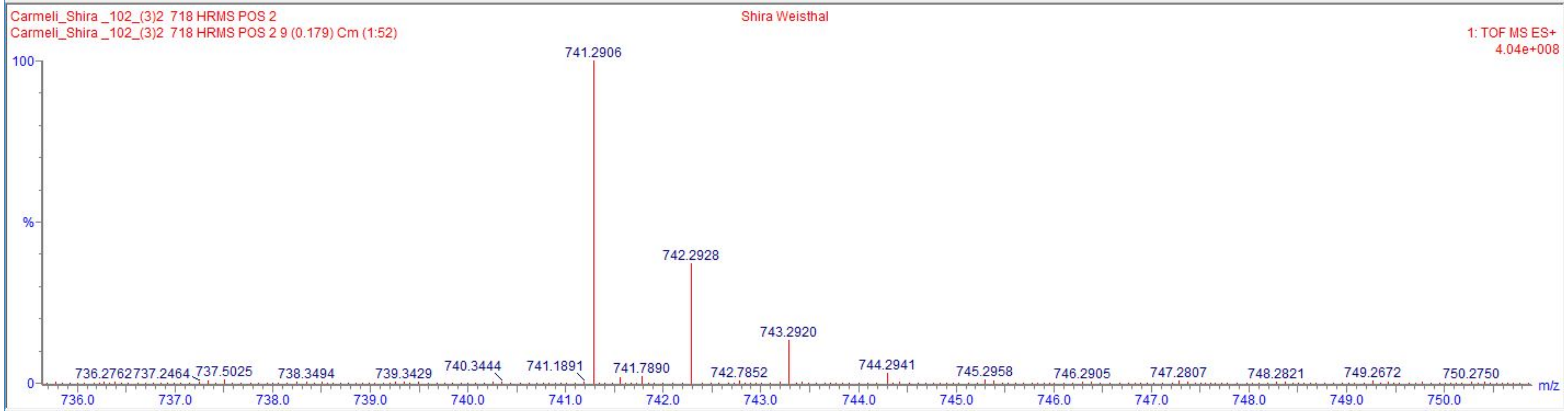

Figure S19. LCMS Traces of Marfey's Method Amino Acids Analysis of Aeruginosin KT718 (**2**)

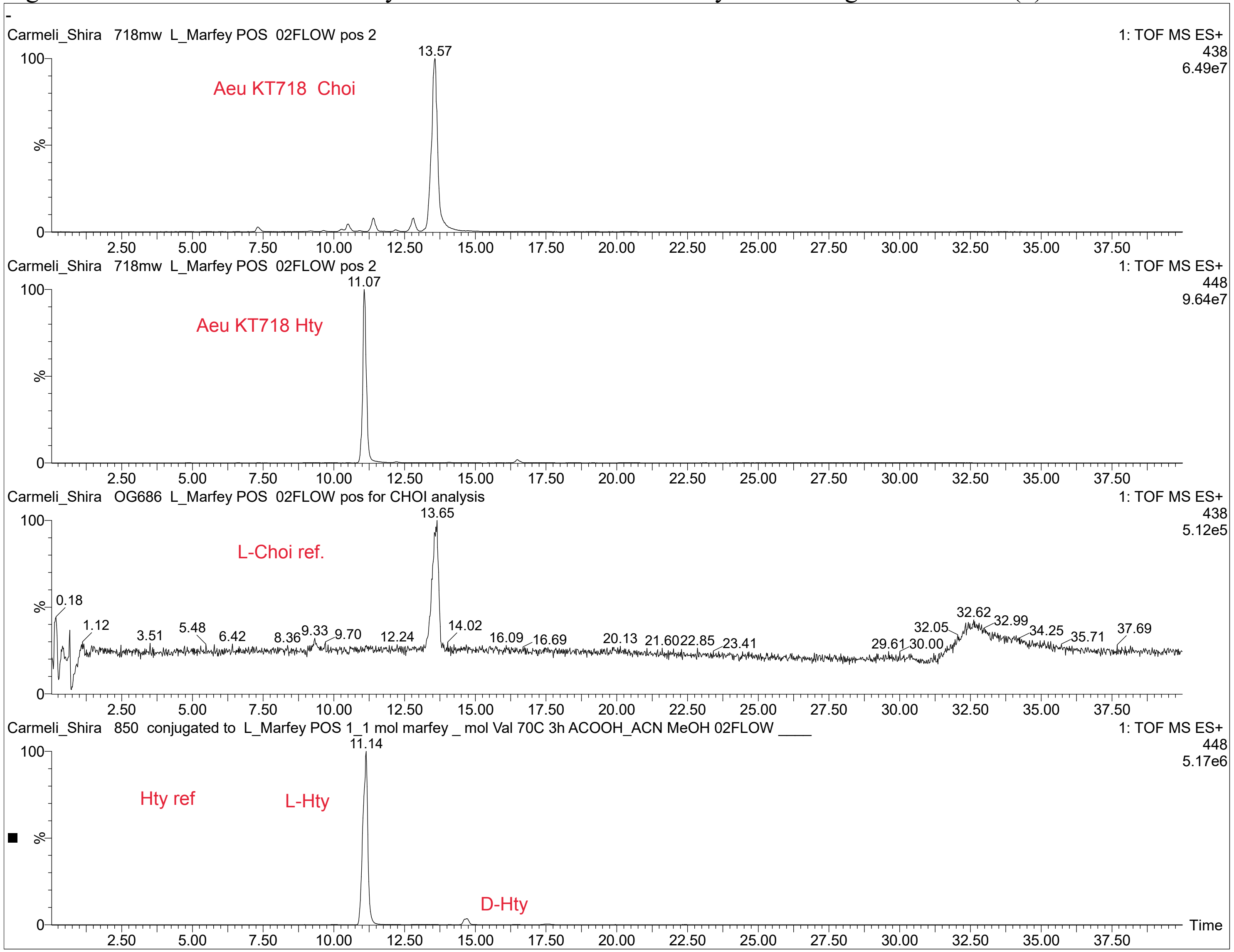

Figure S20. HPLC Traces of Chiral Chromatography of HPLA Analysis of Aeruginosin KT718 (**2**)

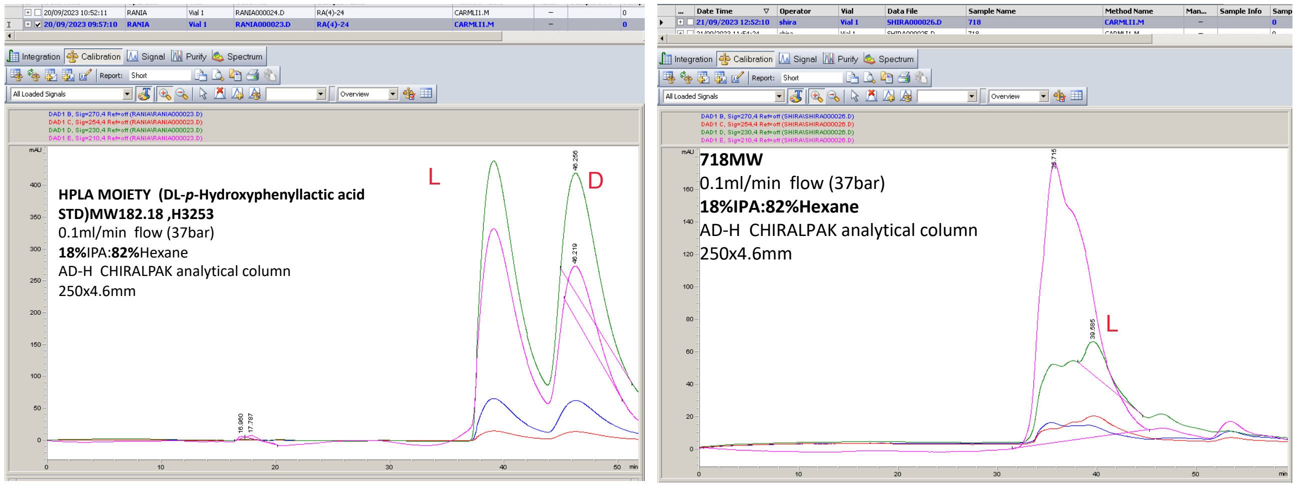

Figure S21. ^{1}H NMR Spectrum of Aeruginosin KT575 (**3**) in DMSO-d_6

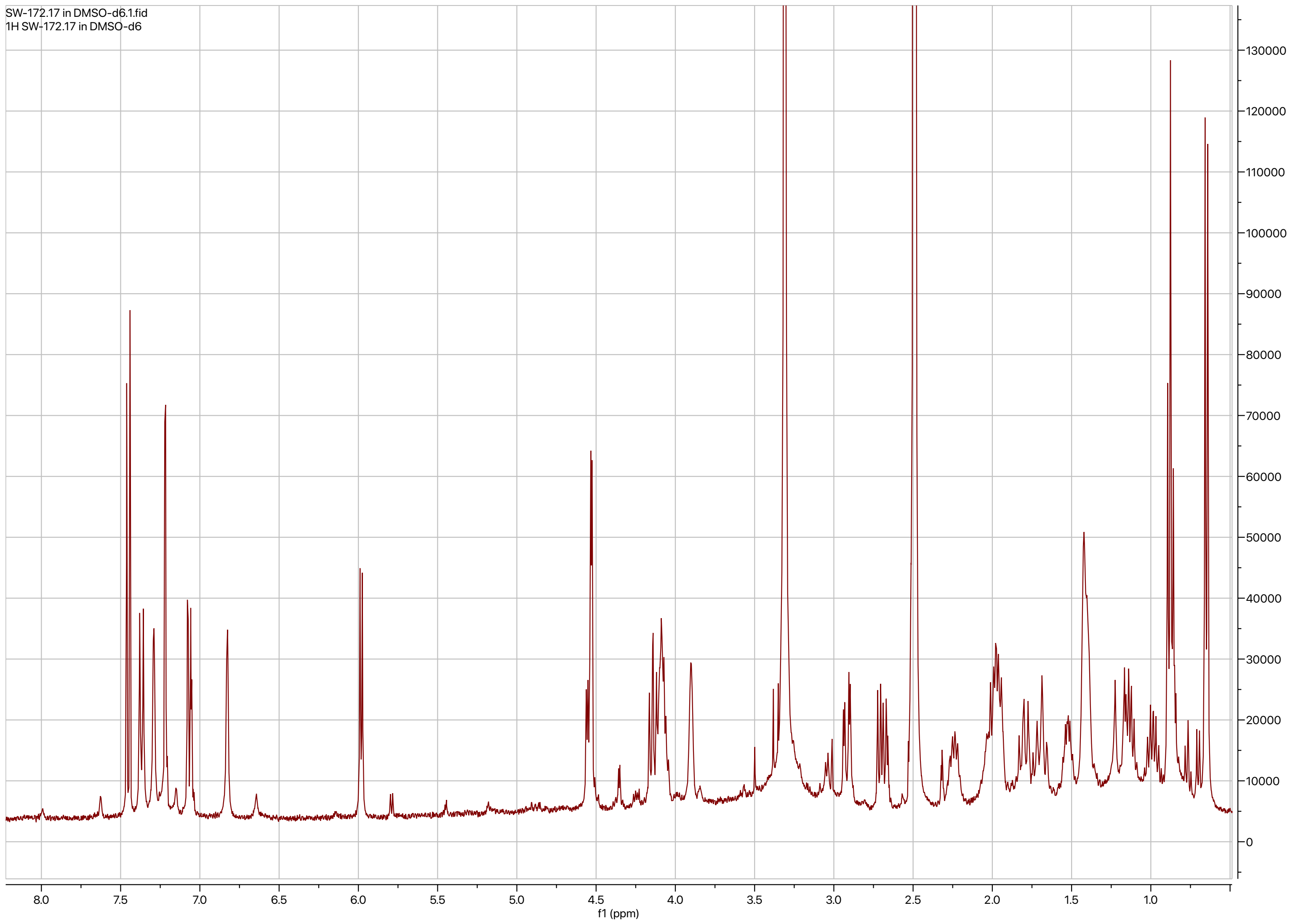

Figure S22. ^{13}C NMR Spectrum of Aeruginosin KT575 (**3**) in DMSO-d_6

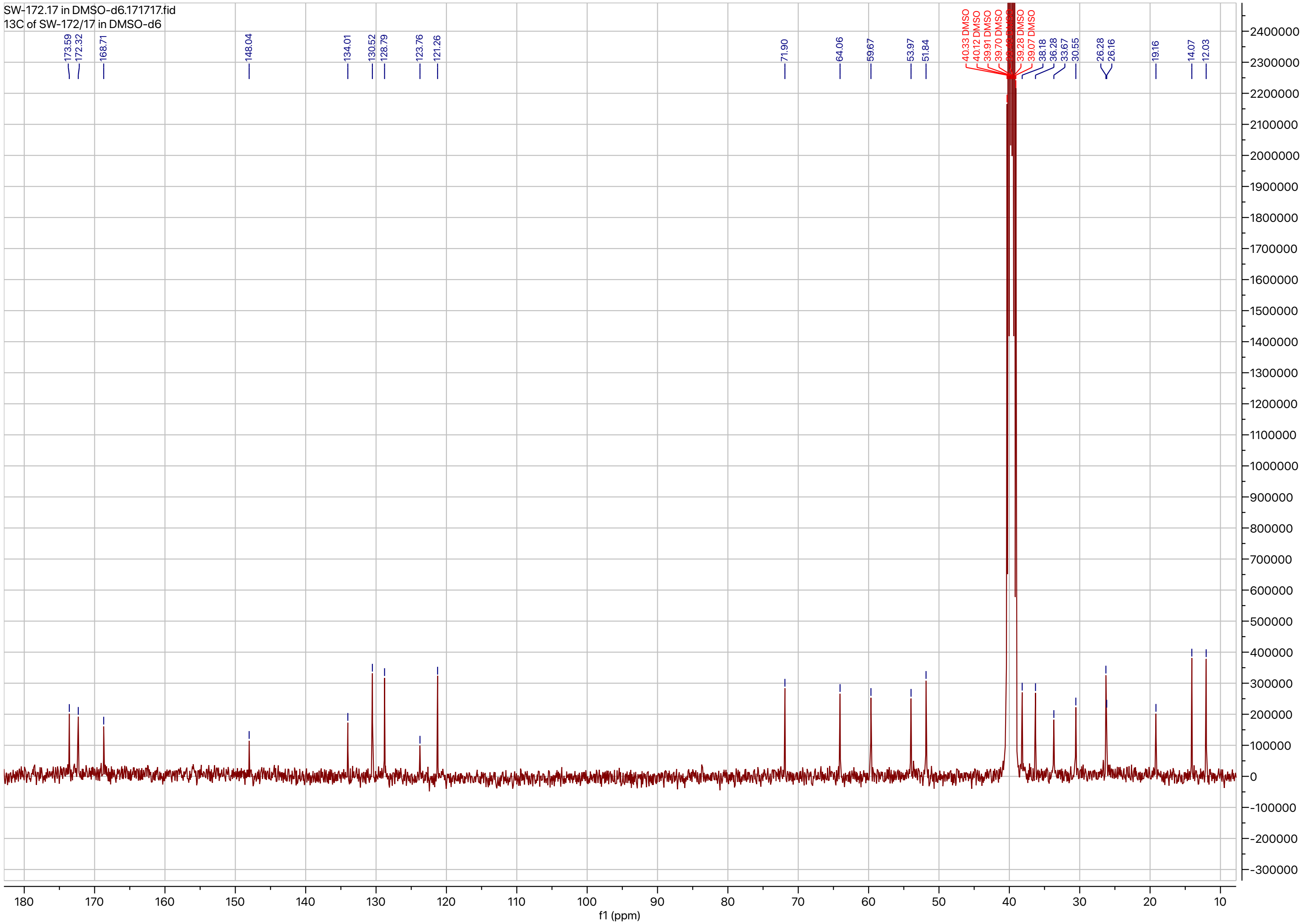

Figure S23. HSQC Spectrum of Aeruginosin KT575 (**3**) in DMSO-d_6

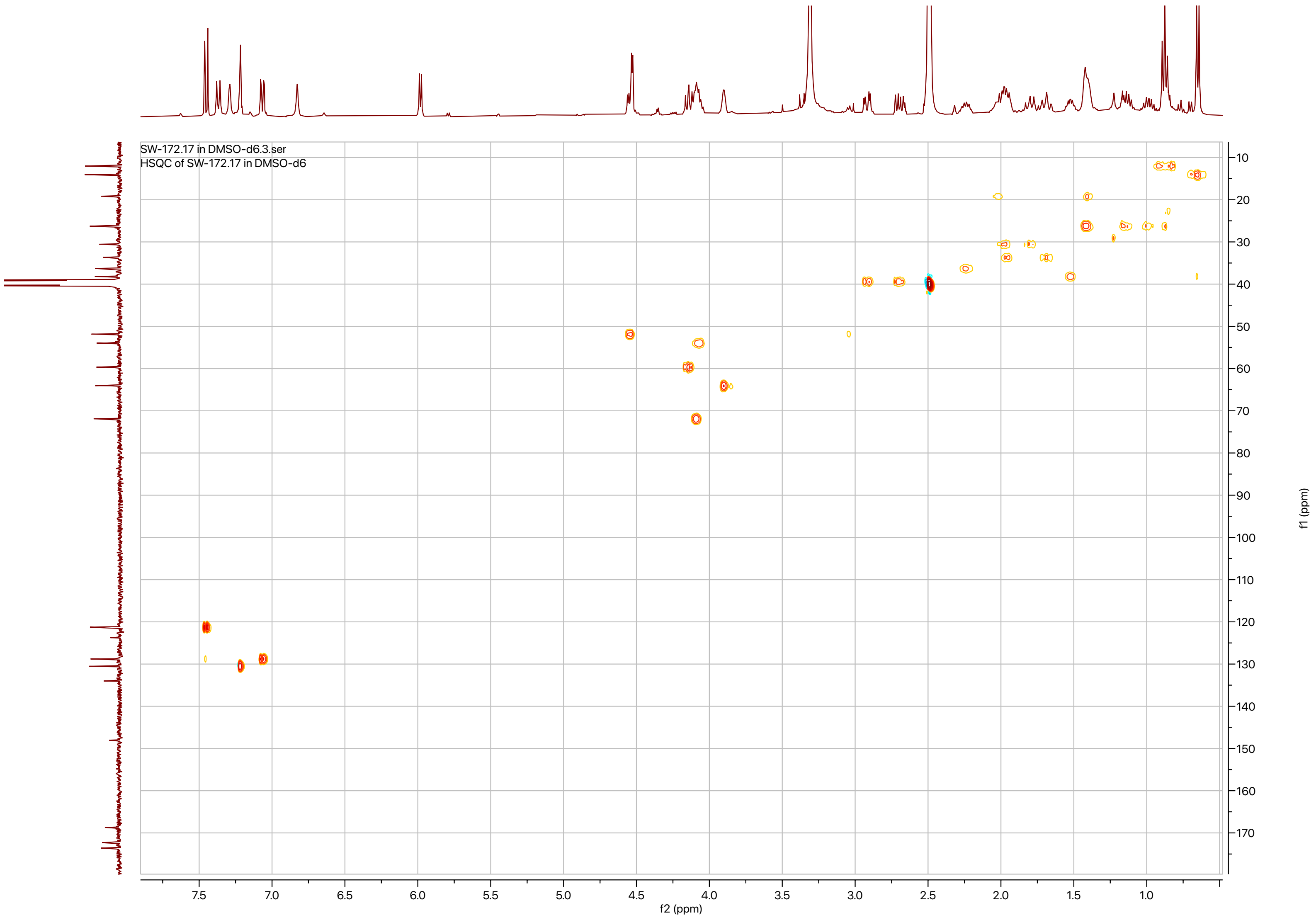

Figure S24. HMBC Spectrum of Aeruginosin KT575 (**3**) in DMSO-d_6

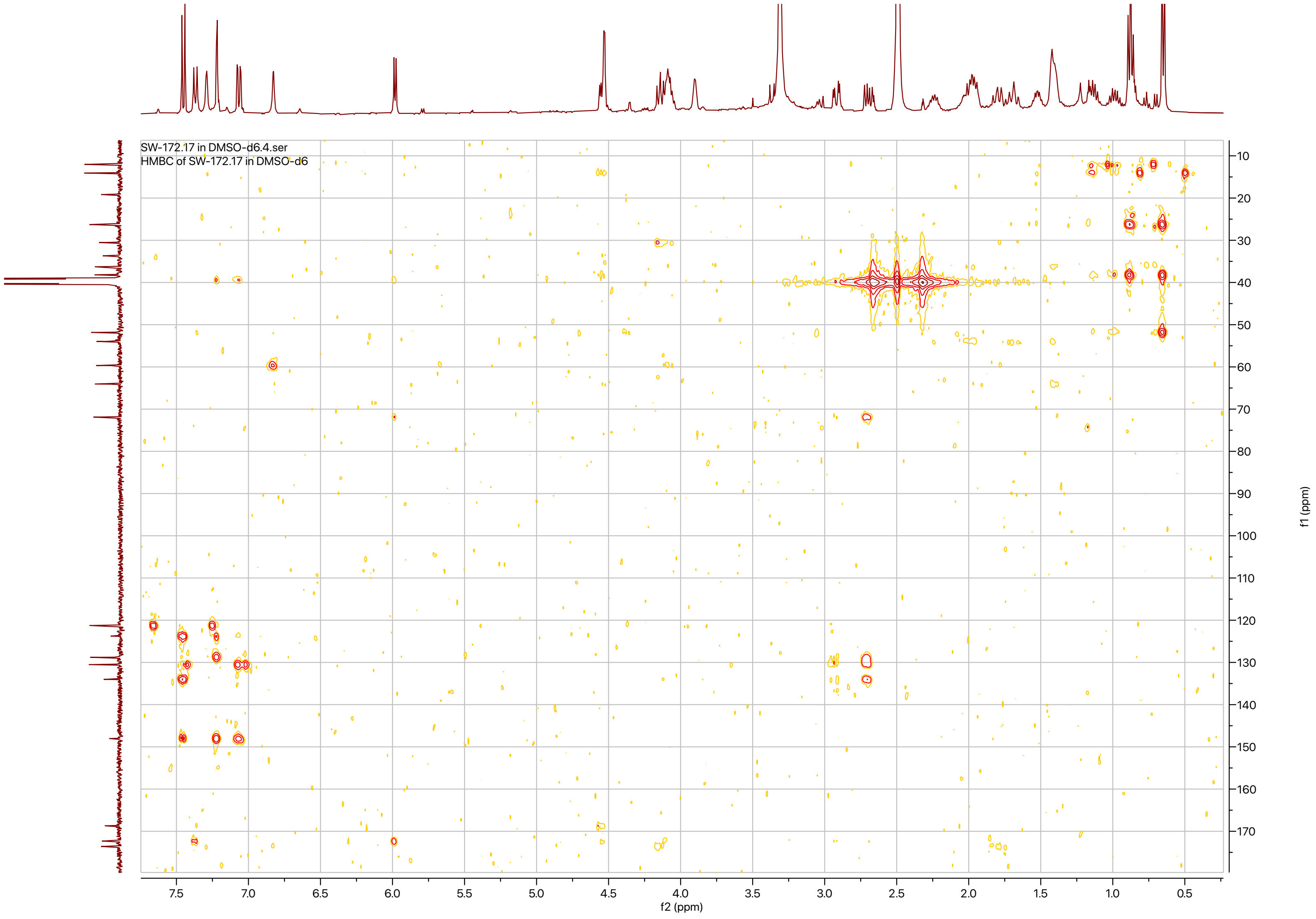

Figure S25. COSY Spectrum of Aeruginosin KT575 (**3**) in DMSO-d_6

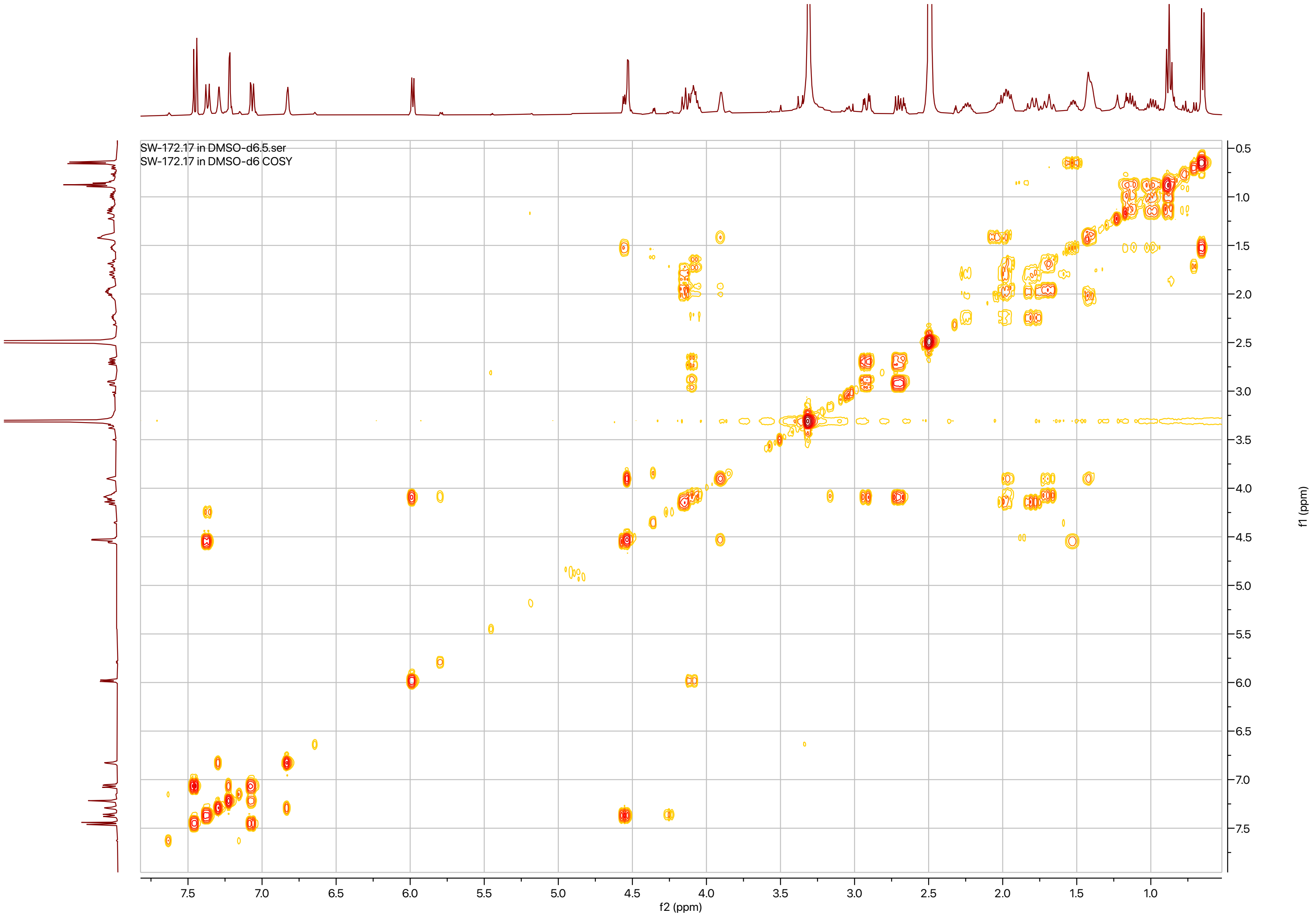

Figure S26. TOCSY Spectrum of Aeruginosin KT575 (**3**) in DMSO-d_6

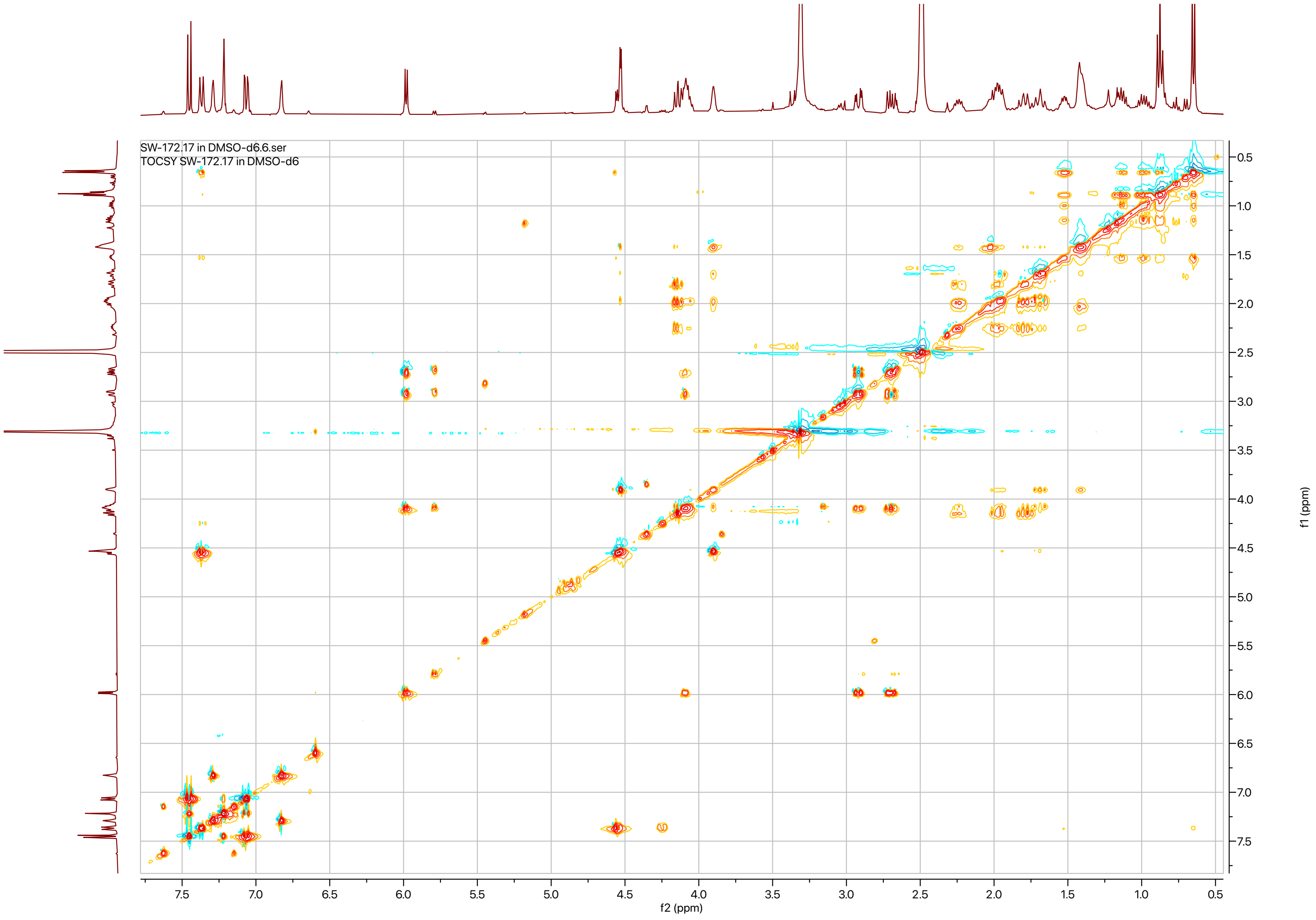

Figure S27. ROESY Spectrum of Aeruginosin KT575 (**3**) in DMSO-d_6

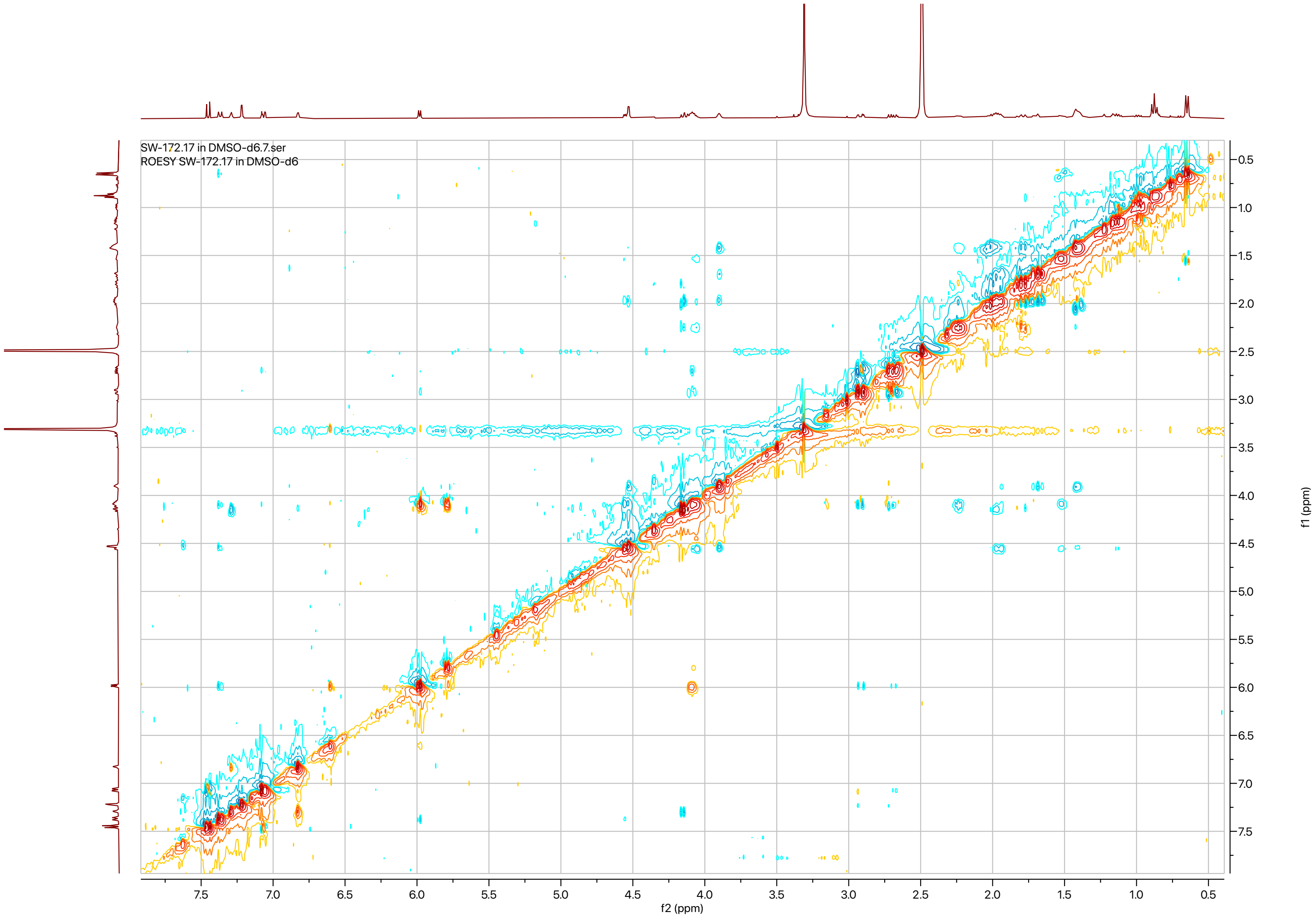

Figure S28. Negative HR ESI MS/MS Spectrum of Aeruginosin KT575 (**3**)

Single Mass Analysis
Tolerance = 10.0 PPM / DBE: min = -1.5, max = 50.0
Element prediction: Off
Number of isotope peaks used for i-FIT = 2
Monoisotopic Mass, Odd and Even Electron Ions
877 formula(e) evaluated with 3 results within limits (all results (up to 1000) for each mass)
Elements Used:

Mass	Calc. Mass	mDa	PPM	DBE	Formula	i-FIT	i-FIT Norm	Fit Conf %	C	H	N	O	S	Cl
574.1647	574.1626	2.1	3.7	9.5	C24 H33 N3 O9 S Cl	570.6	0.246	78.18	24	33	3	9	1	1
	574.1687	-4.0	-7.0	9.0	C24 H35 N4 O6 S2 Cl	572.6	2.247	10.57	24	35	4	6	2	1
	574.1691	-4.4	-7.7	15.0	C24 H27 N8 O7 Cl	572.6	2.184	11.26	24	27	8	7		1

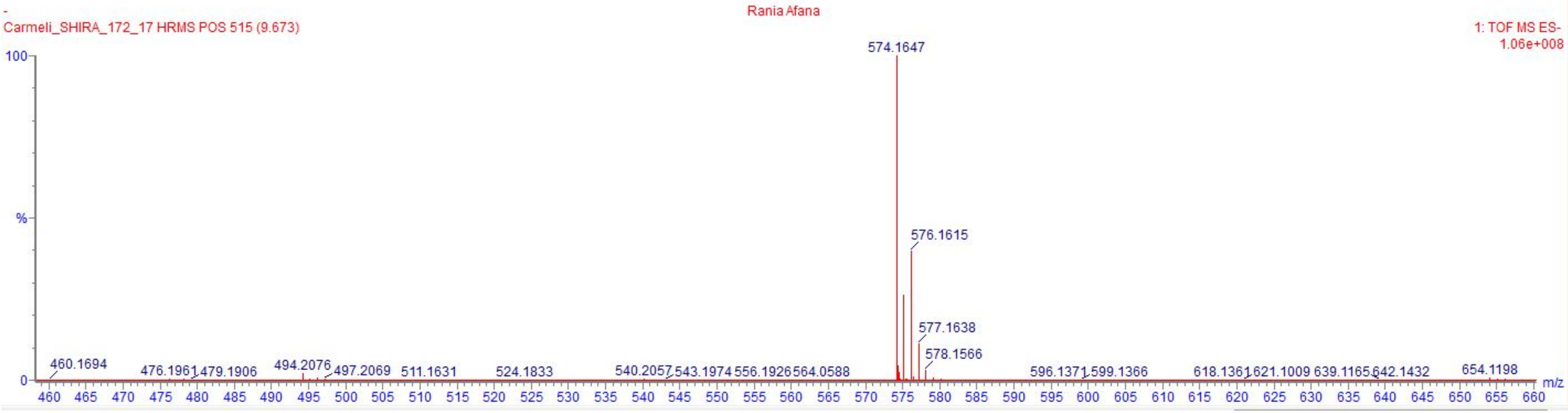

Figure S29. LCMS Traces of Marfey's Method Amino Acids Analysis of Aeruginosin KT575 (**3**)

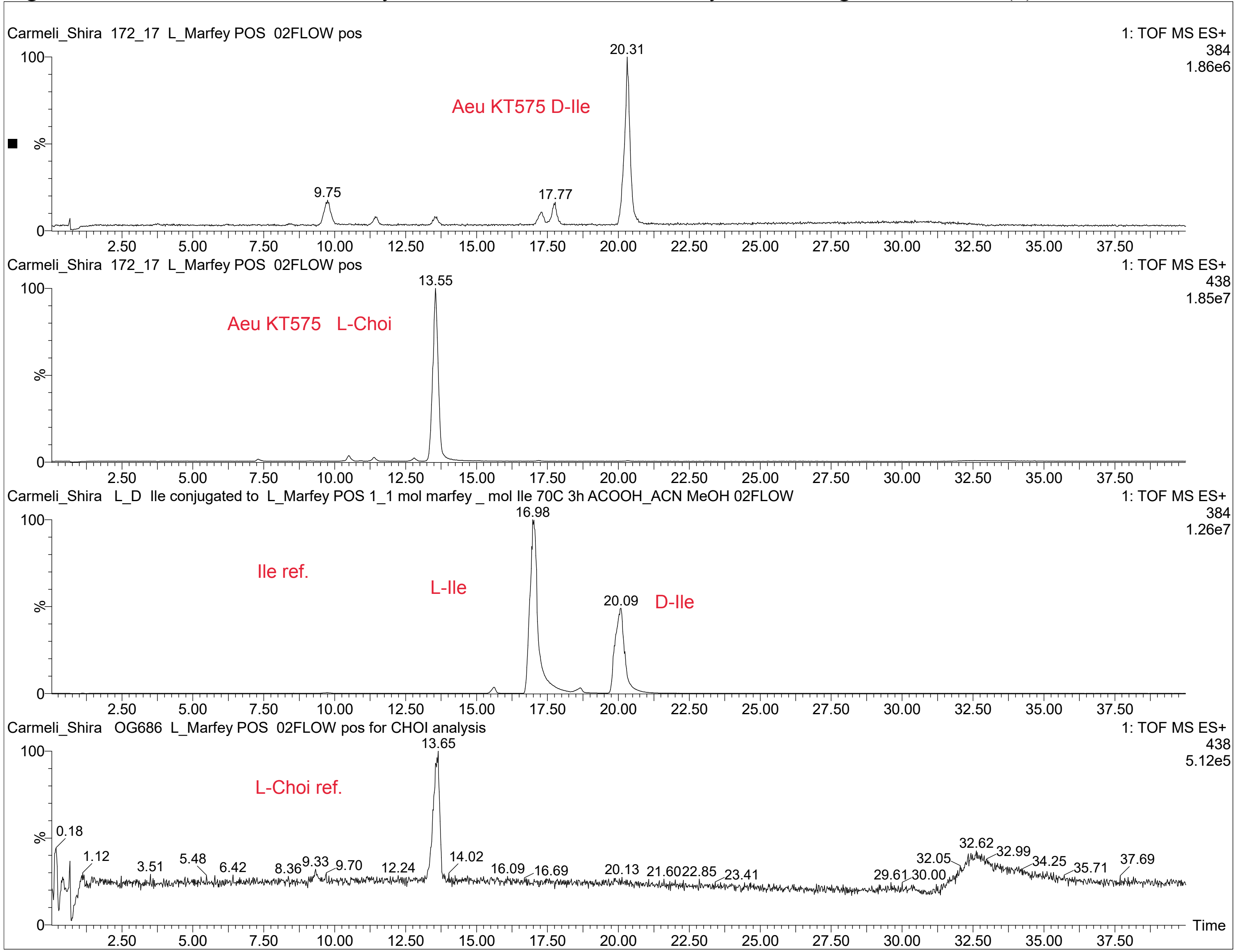

Table S6. Comparison of the ^{1}H and ^{13}C NMR data of the 6-Cl-Hpla-7-sulfate moiety in Aeruginosin KT575 (**3**) and Aeruginosin 89-B in DMSO-d_6

Aeruginosin KT575 (**3**)[a]			Aeruginosin 89-B[b]		
Position	δ_C, Mult.	δ_H, Mult. (J in Hz)	Position	δ_C, Mult.	δ_H, Mult. (J in Hz)
1	172.3, C		1	172.0, C	
2	71.9, CH	4.09, m	2	71.6, CH	4.08, dd (7.3,3.9)
2-OH		5.98, d (5.8)	2-OH		-
3pe	39.4, CH_2	2.92, dd (14.0,3.4)	3a	39.0, CH_2	2.93, dd (13.7,3.9)
3pa		2.70, dd (14.0,7.6)	3b		2.70, dd (13.7,7.3)
4	134.0, C		4	133.8, C	
5	130.5, CH	7.22, d (2.1)	5	130.3, CH	7.20, d (1.7)
6	123.8, C		6	123.6, C	
7	148.0, C		7	147.8, C	
8	121.3, CH	7.45, d (8.9)	8	121.2 CH	7.46, d (8.6)
9	128.8, CH	7.07, dd (8.9,2.1)	9	128.6 CH	7.05, dd (8.6,1.7)

[a]400 MHz for ^{1}H, 100 MHz for ^{13}C. [b]600 MHz for ^{1}H, 150 MHz for ^{13}C.

Table S7. Comparison of the ^{1}H and ^{13}C NMR data of the Choi moiety of Aeruginosin KT575 (**3**) and Aeruginosin DA495B in DMSO-d_6[a]

Aeruginosin KT575 (**3**)			Aeruginosin DA495B		
Position	δ_C, Mult.	δ_H, Mult. (*J* in Hz)	Position	δ_C, mult.	δ_H, mult., *J* (Hz)
Choi-1	173.6, C		Choi-1	173.5, C	
1- NH_2		7.29, d (1.0)	1- NH_2		7.23, s
					6.83, s
		6.83, d (1.0)			
2	59.7, CH	4.14, dd (9.6,8.4)	2	59.7, CH	4.12, dd (9.3,8.2)
3pe	30.6, CH_2	1.97, m	3	30.6, CH_2	1.97, m
					1.79, m
3pa		1.79, ddq (12.5, 9.8, 7.8)			
3a	36.3, CH	2.24, ddq (12.9, 7.1, 6.0)	3a	36.3, CH	2.23, m
4pe	19.2, CH_2	2.02, m	4	19.2, CH_2	2.02, m
					1.39, m
4pa		1.41, m			
5	26.2, CH_2	1.42, m	5	26.1, CH_2	1.40, m
6	64.1, CH	3.90, brs	6	64.0, CH	3.91, brs
6-OH		4.53, d (3.1)	6-OH		4.48, d (2.8)
7pe	33.7, CH_2	1.96, m	7	33.7, CH_2	1.96, m
7pa		1.69, brtd (11.8, 1.9)			1.65, brt (12.0)
7a	54.0, CH	4.07, m	7a	54.1, CH	4.00, ddd (11.2, 6.5,5.9)

[a]400 MHz for ^{1}H, 100 MHz for ^{13}C.
